# Beyond the chemical master equation: Stochastic chemical kinetics coupled with auxiliary processes

**DOI:** 10.1371/journal.pcbi.1009214

**Published:** 2021-07-28

**Authors:** Davin Lunz, Gregory Batt, Jakob Ruess, J. Frédéric Bonnans

**Affiliations:** 1 Inria Saclay – Île de France, Palaiseau, France; 2 École Polytechnique, CMAP, Palaiseau, France; 3 Inria Paris, Paris, France; 4 Institut Pasteur, Paris, France; Ecole Polytechnique Fédérale de Lausanne, SWITZERLAND

## Abstract

The chemical master equation and its continuum approximations are indispensable tools in the modeling of chemical reaction networks. These are routinely used to capture complex nonlinear phenomena such as multimodality as well as transient events such as first-passage times, that accurately characterise a plethora of biological and chemical processes. However, some mechanisms, such as heterogeneous cellular growth or phenotypic selection at the population level, cannot be represented by the master equation and thus have been tackled separately. In this work, we propose a unifying framework that augments the chemical master equation to capture such auxiliary dynamics, and we develop and analyse a numerical solver that accurately simulates the system dynamics. We showcase these contributions by casting a diverse array of examples from the literature within this framework and applying the solver to both match and extend previous studies. Analytical calculations performed for each example validate our numerical results and benchmark the solver implementation.

## Author summary

Populations of genetically identical cells tend to exhibit remarkable variability. This seemingly counter-intuitive observation has broad and fascinating implications, and has thus been a focal point of biological modeling. Many important processes act on this cellular heterogeneity at the population level, leading to an intricate coupling between the single-cell and the population-level dynamics. For example, selection pressures or growth rates may depend crucially on the expression of a particular gene (or gene family). Classical single-cell modeling approaches, such as the chemical master equation, can accurately describe the mechanisms driving cellular noise, however, they cannot encapsulate how the aforementioned auxiliary processes affect the population composition. In this work, we propose a unifying framework that extends the classical chemical master equation to faithfully capture the single-cell variability alongside the population-level evolution. We develop, analyse, and showcase an open-source numerical tool to simulate the dynamics of such populations in time. The tool is designed for straightforward use by a non-technical audience: a high-level description of the underlying chemical and population-level processes suffices to simulate complex system dynamics. Simultaneously, we retain high customisability of the underlying mathematical representation for the more advanced user. Ultimately, the unifying framework and the associated computational tool open new horizons in the study of how fundamental microscopic dynamics give rise to complex macroscopic phenomena.

This is a *PLOS Computational Biology* Methods paper.

## Introduction

**Fig 1:**
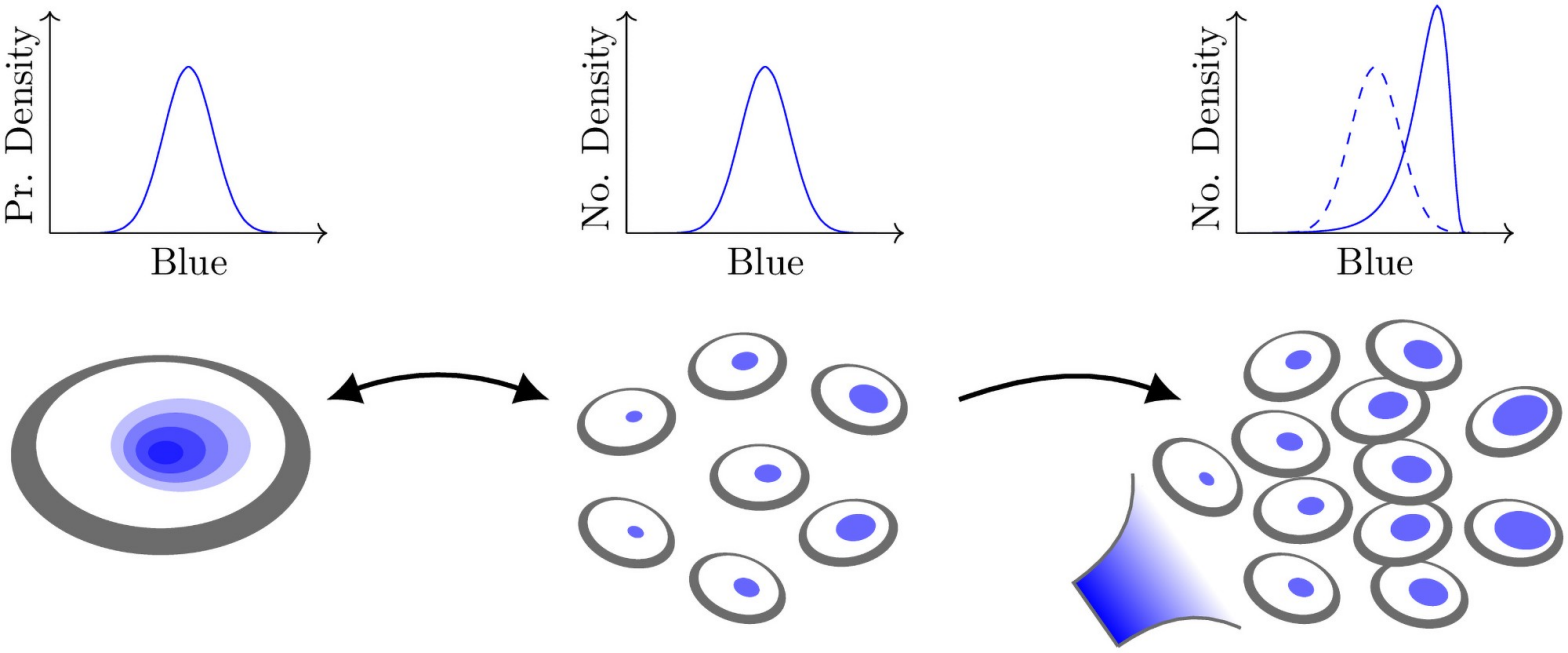
Schematic depiction of an auxiliary process modifying the population-level density. The measurable concentration of a nominal “blue” chemical species within a cell that is the downstream result of some chemical reaction network. The probability density of concentration in a single cell (left) is identical to the number density in a large population of identical cells (centre). However, when a state-dependent pressure (such as amplified growth for higher blue concentration) is exerted on the population (right), the distribution is affected in a way that may not be encapsulated by a classical CME. In this paper, we study a framework in which chemical reaction networks are coupled to such auxiliary processes, and we introduce a software package to simulate the associated dynamics.

The chemical master equation (CME) [1] governs the evolution of the probability distribution of the configuration, or state, of a reaction network. Typically, the configuration describes the number of molecules of various species, such as chemical reactants undergoing reactions. The CME and its continuum approximations, in particular the Fokker–Planck approximation [2], are fundamental modeling tools used for describing chemical reaction networks. These reaction networks find broad application across the quantitative sciences, providing accurate descriptions of a wide variety of chemical, biological and social phenomena [3]. Crucially, this family of models accounts for stochasticity inherent in reaction processes that play functional roles in biochemical contexts [4, 5]. In a population of individuals each governed by identical reaction kinetics, such as gene expression in a population of cells, this stochasticity manifests in cell-to-cell variability. When a state-dependent selection pressure is exerted upon the population, for example, phenotypic growth or cell-fate decisions, individuals are affected heterogeneously due to this cell-to-cell variability [6, 7]. The dynamics of such phenomena cannot be captured simply by a stochastic reaction network [8]. Models that incorporate population-level selection may break detailed balance and introduce nonlinearity into the master equation [9], rendering explicit solutions unknown in all but the simplest of circumstances [10]. We depict the example of state-dependent growth in a population of cells in Fig 1.

Recently this problem was addressed by Duso and Zechner [11] by tracking the number of cells with each possible internal state (as opposed to tracking only the state of a single cell). Biochemical reactions driving a single cell to transition from one state to another are encapsulated by an associated change to the population count: one fewer in the old state, one more in the new state. While the total population is preserved under such transitions, changes to the population composition, such as the introduction or removal of cells, may be included by laws governing non-conservative counter increments or decrements. This formalism is equivalent to a Markov chain on the state space of mappings from internal configurations to the natural numbers. While conceptually appealing, such a state space is an exponential inflation of the single-cell state space. For example, the dynamics of fewer than 100 molecules in each cell (of just a single species) in fewer than 50 cells, corresponds to a state space of size 10^100^, far more than the number of known atoms in the universe. This renders calculations of the full distribution infeasible, and motivates the moment closure approximations studied by Duso and Zechner [11].

In this study, we develop a modeling framework that reaches beyond the classical CME to incorporate such population-level processes while effectively preserving the state space of the single-cell Markov chain. Keeping in mind that when the typical number of a species is large the CME is challenging to solve, we allow a continuum approximation in the form of the Fokker–Planck equation. We also allow non-local transitions to incorporate phenomena discontinuous in state such as cell division and fragmentation [12, ch. 4], as well as phenomena where production machinery operates on timescales significantly shorter than the timescale of interest [13], such as in protein production in “bursts”. This class of models finds application beyond biochemical physics, such as in communications protocols [14].

Crucially, not all species need be cast in the continuum, and thus we retain a hybrid structure: species of typically large number are in the continuum, while the remaining species retain a discrete description. This discrete structure allows us not only to capture discrete molecule numbers, but also to model abstract system states. For example, we may consider transcription factors in gene expression in a bound or unbound state, or distinguish cells based on phases of the cell cycle, as well as cell-fate decisions such as differentiation and recombination. This demonstrates the importance of retaining a discrete structure in the framework, which allows us to capture both discrete molecule numbers, as well as discrete abstract states. In this study, we leverage the discrete structure to capture first-passage times [15], providing insights that lend a new and extended perspective to existing results.

Some population-level processes may be resolved by leveraging this same discrete structure in non-physical ways. In this case, the discrete states are not simply abstract states, as they may no longer correspond to physical configurations at all. For example, we may need to consider negative “reaction” rates, breaching the physical description of a classical chemical reaction network. We highlight the distinction by calling these phantom states. By constructing systems incorporating phantom states, we show that we are able to couple CME dynamics to non-standard processes by casting them within our general framework.

Finally, we show how other classes of auxiliary processes fall within this framework, such as growth–fragmentation processes used to describe cell growth and division, as well as several other physical phenomena.

Our approach is conceptually akin to that pursued by Thomas [7], whereby averaging stochastic fluctuations due to small population sizes allows us to track the population-density evolution on the original state space. This approach is very useful for large populations where such fluctuations may be neglected. We generalise the class of problems studied in Thomas [7] by including a continuum formulation for internal states, allowing state-dependent rates, and resolving transient distributions. For small population sizes where stochastic fluctuations must be accounted for, the framework developed by Duso and Zechner [11] is more appropriate.

With the goal of open and collaborative progress, we make our code publicly available [16] in the hope that this modeling tool can be of broader use for the community. The code is generic and has been written for arbitrary reaction networks, growth–fragmentation and similar models. The interface is intended to be straightforward for the first-time user, while also allowing more advanced control of the numerical scheme, including discretisation and time-stepping details.

The rest of the paper is structured as follows. First, we introduce the encompassing framework by defining the class of problems under consideration. We then outline the numerical software developed to solve problems cast in this framework. The results are presented as a series of case studies of auxiliary processes not classically captured by the CME: a first-passage time problem in self-regulated gene expression, a birth–death process under population-wide growth pressure, and a growth–fragmentation model. Finally, we discuss the results and their implications. For the sake of completeness, in Section 1 in S1 Appendix we include an extensive description of the numerical scheme implemented in the software.

## Methodology

### Augmented CME framework

We consider a stochastic reaction network of *d* species, which we write in vector form ***X*** ≔ (*X*_1_, *X*_2_, …, *X*_*d*_)^*T*^, undergoing reactions labeled by i∈I, which we write as

$$\begin{array}{c} c_{i}\cdot X\;\overset{\;R_{i}\left(X,t\right)\;}{\to }\;d_{i}\cdot X, \end{array}$$ (1)

for reactant species and quantities determined by $c_{i}\in Z^{d}$ and product species and quantities determined by $d_{i}\in Z^{d}$. The reaction rate *R*_*i*_ may depend on the system state and time. The system state is a continuous-time Markov chain and may be described by its law, *P*(***X***, *t*), the probability of being in state ***X*** at time *t*. The chemical master equation (CME), governing the evolution of the law [1], takes the form

$$\begin{array}{c} \frac{\partial P(X,t)}{\partial t}=\sum_{i\in I}R_{i}\left(X+c_{i}-d_{i},t\right)P\left(X+c_{i}-d_{i},t\right)-R_{i}\left(X,t\right)P\left(X,t\right). \end{array}$$ (2)

We highlight a gentle abuse of notation: ***X*** denotes species labels in the reaction network description (1), but is an independent variable in $Z^{d}$ in the law in (2). Only the latter will be used henceforth as we focus on the law of the stochastic process.

We assume that the species typically occur in large numbers, and seek to approximate the discrete description on the continuum. Defining ***e***_*i*_ ≔ ***d***_*i*_ − ***c***_*i*_, the Fokker–Planck approximation [17, Ch. 5] is given by

$$\begin{array}{c} \frac{\partial }{\partial t}p\left(x,t\right)=\sum_{i\in I}-e_{i}^{T}\nabla \left[r_{i}\left(x,t\right)p\left(x,t\right)\right]+\frac{1}{2\Omega }e_{i}^{T}\vec{H}\left(r_{i}\left(x,t\right)p\left(x,t\right)\right)e_{i}, \end{array}$$ (3)

where Ω ≫ 1 is the system size, ***x*** = ***X***/Ω, *r*_*i*_ = *R*_*i*_/Ω and $\vec{H}$ denotes the Hessian. The probability mass *P*(***X***, *t*) is approximated by *p*(***x***, *t*)/Ω^*d*^. Eq (3) is a particular form of the more general Fokker–Planck equation [18].

We may extend the class of reactions by modeling non-local state changes to describe, for example, species production in “bursts”. We emphasise that we use the terms “non-local” and “local” with respect to the state; spatial location is not accounted for in this study. Such a process, indexed by j∈J, may be associated with a kernel $B_{j}\left(y,t\right)$, typically describing the probability density of a state change of size y∈R at time *t*, and the rate *f*_*j*_(***x***, *t*), describing the rate of such changes occurring from state ***x*** at time *t*. The non-local model then takes the form

$$\begin{array}{c} \begin{array}{cc} & \frac{\partial }{\partial t}p\left(x,t\right)=\sum_{i\in I}-e_{i}^{T}\nabla \left[r_{i}\left(x,t\right)p\left(x,t\right)\right]+\frac{1}{2\Omega }e_{i}^{T}\vec{H}\left(r_{i}\left(x,t\right)p\left(x,t\right)\right)e_{i}\\ & \;+\sum_{j\in J}-f_{j}\left(x,t\right)p\left(x,t\right)+∥e_{j}∥\int_{x-ze_{j}\in R_{+}^{d}}f_{j}\left(x-ze_{j},t\right)p\left(x-ze_{j},t\right)B_{j}(z∥e_{j}∥,t)\;dz. \end{array} \end{array}$$ (4)

This is also closely related to droplet breakup and coalescence, where the equation is called the Smoluchowski coagulation equation, and has been used extensively in models from aerosols to cosmological structure formation [19]. While our focus is on the breakup process rather than coalescence (or aggregation), this framework may be extended to include such terms.

Lastly, we introduce discrete states indexed by k∈K, whereby the probability is distributed among the family of densities ${\left\{p_{k}\right\}}_{k\in K}$. There is a flux of probability from state k∈K to state ℓ∈K at a rate of *g*_*kℓ*_(***x***, *t*), that may depend on the continuum state and time. Importantly, all other rates (reaction and non-local) may depend on the discrete state *k*, and are subscripted to reflect this. In this way, we avoid having to consider reactions and non-local processes, indexed by I and J, respectively, that depend on the discrete state k∈K. Instead, we may consider I and J to include the union of all local and non-local interactions, and set the rates to zero for the appropriate k∈K. The final form of the system is

$$\begin{array}{c} \begin{array}{cc} & \{\frac{\partial }{\partial t}p_{k}\left(x,t\right)=\sum_{i\in I}-e_{i}^{\;T}\nabla \left[r_{ik}\left(x,t\right)p_{k}\left(x,t\right)\right]+\frac{1}{2\Omega }e_{i}^{\;T}\vec{H}\left(r_{ik}\left(x,t\right)p_{k}\left(x,t\right)\right)e_{i}\\ & \;+\sum_{j\in J}-f_{jk}\left(x,t\right)p_{k}\left(x,t\right)+∥e_{j}∥\int_{x-ze_{j}\in R_{+}^{d}}f_{jk}\left(x-ze_{j},t\right)p_{k}\left(x-ze_{j},t\right)B_{jk}(z∥e_{j}∥)\;dz\\ & \;+\sum_{\ell \in K}-g_{k\ell }\left(x,t\right)p_{k}\left(x,t\right)+g_{\ell k}\left(x,t\right)p_{\ell }\left(x,t\right){\}}_{k\in K}. \end{array} \end{array}$$ (5)

We henceforth call system (5) the Augmented Chemical Master Equation (ACME). The ACME framework describes a hybrid model that generalises the continuum Fokker–Planck equation (the first line of (5)) and the discrete CME (the last line of (5)) by encapsulating both descriptions simultaneously alongside non-local transitions of the continuum state (the second line of (5)). Conceptually, the continuum components were motivated by the need for efficient approximations of the CME for large state spaces, and they typically preserve the underlying master equation structure. Therefore, the simplest conception of the ACME framework remains fundamentally classical; describing the evolution of the probability of a single cell to have a particular internal state. This begs the question: in what sense, beyond merely piecing together the hybrid formulation, is the ACME augmenting the CME? We answer this question below by discussing two technical assumptions made in this section that may be relaxed. The relaxed assumptions violate the classical framework, but in so doing allow the ACME framework to reach beyond the CME in physically meaningful ways, which we demonstrate with concrete example applications.

### The augmentation and its interpretation

It is instructive to first contemplate what the discrete states model. Retaining a discrete description is crucial for molecules not expected to be present in large numbers. In this case, we consider *d* to denote the number of continuum species. The discrete states may also be used to describe abstract system states in which different behaviour governs the reaction network. For example, light-induced recombination [20] allows cells to alter their genetic structure, thereby changing the reaction network behaviour. This transition to a different governing dynamic may be encapsulated by transition between discrete states. A third use-case arises when discrete transition rates are negative, whereby some discrete states no longer correspond to a physical system configuration: the density may become negative and thus no longer corresponds to a probability measure. We will show that these phantom states enable a broader class of auxiliary population-level dynamics to be encapsulated as the model retains a physically meaningful interpretation.

The non-local contributions introduced above are inspired by long-range changes of cell state, such as production in large bursts. We will demonstrate that particular jump kernels that are not probability distributions violate classical conservation properties. Nevertheless, these can simultaneously allow us to capture certain auxiliary population-level processes and thus the ACME framework remains a useful population description.

The ACME framework augments the classical framework in violating these two classical assumptions—that the discrete transition rates *g*_*kℓ*_ are non-negative, and that the jump kernels $B_{jk}$ are probability densities. The result is that the density described by the ACME framework may no longer describe the probability distribution of an underlying stochastic process of a single cell, since the density may have negative components or be non-conservative (and thus not sum to unity). Instead, it captures the evolution of the prevalence of different states within a population. In other words, the interpretation of a probability density is replaced by that of a population density. Such populations are governed not only by the changes of internal state of single cells, but additionally by auxiliary processes, those that act to change the composition of the population. In this way, the positive components may still describe a physically valid population density and they need not be conservative. This paradigm shift is illustrated in Fig 1: the vertical axes on the left panel (classical CME conception) describe probability densities while on the right (ACME framework) they describe number densities.

It is worth classifying which auxiliary processes are incorporated in this formulation. Unary auxiliary processes—those originating from a single individual—are incorporated. This includes the duplication or removal of individuals, e.g. due to growth or death of a single cell, as well as the redistribution of a single cell, e.g. cell division. Nullary processes—those not originating from the existing population, such as external introduction of new individuals into the population—may also be incorporated via abstract states. Binary and higher-order processes, such as multiple cells fusing, are not presently included.

Equipped with the ACME framework of system (5), we proceed to outline the implementation of a numerical solver capable of solving system (5) in the general case, which we call the Flips solver.

### The Flips library

In this section we briefly survey numerical solvers in the chemical kinetics literature to establish the context of the numerical scheme we employ. We stress that all of these solutions remain grounded within the classical CME framework, and thus fall short from the population-level perspective.

Numerical simulation of stochastic reaction networks has an extensive and richly developed literature. Rather than an exhaustive survey, we provide an outline of the approaches and their motivations and relative merits. Reaction networks are commonly modeled as a continuous-time Markov chain (CTMC) whose state distribution is governed by the CME. When the possible number of states becomes large, solving the master equation becomes prohibitively challenging. An alternative to solving the CME is to generate exact sample trajectories of the stochastic process via the Stochastic Simulation Algorithm (SSA) [21, 22]. Stochastic simulation of the underlying CTMC is equivalent to the master equation in the sense that each simulated trajectory is a sample from the distribution given by the solution of the master equation. In other words, the underlying stochastic process is identical, the CME captures the full distribution while the SSA merely samples from it. For accurate resolution of the state distribution, large number of sample paths are often required, which has led to a rich set of refinements [23] and approximations [24]. Hybrid approaches that attempt to efficiently capture dynamics at different state [25] or time [26] scales remain a topic of active research. Ultimately, producing a sufficient number of trajectories to accurately resolve the distribution continues to be a challenge.

In an attempt to avoid stochastic sampling but accelerate solution of the full CME, several model reduction approaches have been considered, such as the Finite State Projection method [27], leveraging timescale separation [28], lumping (or aggregating) states [29, and references therein], as well as various spectral approximations [30, 31]. Hybrid combinations of these techniques have also been composed [32, and references therein].

One prevalent model-reduction technique is the class of continuum approximations of the CME, in particular, the Fokker–Planck equation [17, 18]. The underlying assumption is that, when only a small number of molecules need to be simulated the computational complexity is manageable, but when species are expected to have large copy numbers, a continuum approximation is appropriate. Just as the Fokker–Planck equation is the continuum analogue of the CME, the Langevin equation is the continuum analogue of the continuous-time Markov chain. That is, the Langevin equation describes the underlying stochastic process whose distribution is captured by the Fokker–Planck equation.

Various numerical schemes for solving the Fokker–Planck approximation of the CME appear in the literature [33, 34]. Just as with the trajectory sampling, many studies adopt hybrid approaches. In Haseltine and Rawlings [35] and Salis and Kaznessis [36] reactions are partitioned into fast and slow, and sample paths are generated by the Langevin equation or variants of the SSA discussed above. However, the Monte Carlo sampling requires a large number of samples just as for the aforementioned discrete sampling. In Safta et al. [37], the dynamics for each species are partitioned depending on molecule count. A discrete description is implemented for small molecule count (where the continuum assumption breaks down), while the continuum approximation is adopted for larger numbers of molecules. In Sjöberg [38], species are separated into those that are expected to have only small copy numbers for which the discrete description is retained, and those expected to exist in large number where the continuum description is accurate, although non-local contributions in the continuum species are not incorporated.

As we will discuss, in the extended setting of the ACME framework, the quantity *p* governed by (5) does not always represent a probability distribution. Therefore, the direct link to the underlying stochastic process is severed, rendering stochastic sampling techniques not directly applicable. We instead develop a solver based on the system of differential equations (5), which we call the Flips solver (loosely acronymous of the Fokker–Planck system it is designed to solve). Species are separated into those whose description remains discrete, and those whose description are approximated on the continuum, in which we allow non-local dynamics. Most substantially, the solver is built to capture the augmentation of the classical chemical reaction structure, capable of encapsulating auxiliary population-level processes and thus appreciably expanding the model scope, as we demonstrate in the sequel.

To solve system (5) numerically, we begin by discretising the state space. Despite having *d* continuum-state dimensions, each continuum reaction i∈I in the model (5) acts to advect and diffuse probability density only in the ***e_i_*** direction. Since $e_{i}\in Z^{d}$, there is a natural discretisation for an arbitrary network on uniform grids that preserves this one-dimensional structure. Similarly, each burst production changes the state by $e_{j}\in Z^{d}$ for j∈J, and thus the non-local integral term describes an exchange of probability in the ***e***_*j*_ direction, which is similarly preserved on the uniform grid. This observation means that the differential and integral operators act in one-dimension even in a high-dimensional system with multiple species thereby inducing no spurious diffusion in orthogonal directions.

Our approach is to adopt a uniform grid in state space, where we truncate the space at some sufficiently large boundary where we expect only negligible probability density to accumulate. For such a numerical scheme to yield computational advantage over solving the underlying discrete system (2), the state step Δ*x* must satisfy two competing constraints. On the one hand, Δ*x* ≪ 1 is to be sufficiently small so that the scheme is accurate, while on the other hand, it must be nominally larger than a single discrete molecule, that is, Δ*x* > 1/Ω, where Ω is a typical number of molecules (see the scalings below (3)). In practice, the molecule copy number is typically Ω ≳ 10^4^, while we will consider Δ*x* on the order of $O(10^{-2})$, thus both of these constraints are well met.

The conservative finite-volume numerical scheme, based on Kurganov and Tadmor [39], is presented and analysed in Section 1 in S1 Appendix. The Flips solver is made open source [16] to encourage its use and development. We emphasise that the Flips solver has been written with two distinct types of user in mind.

**Fig 2:**
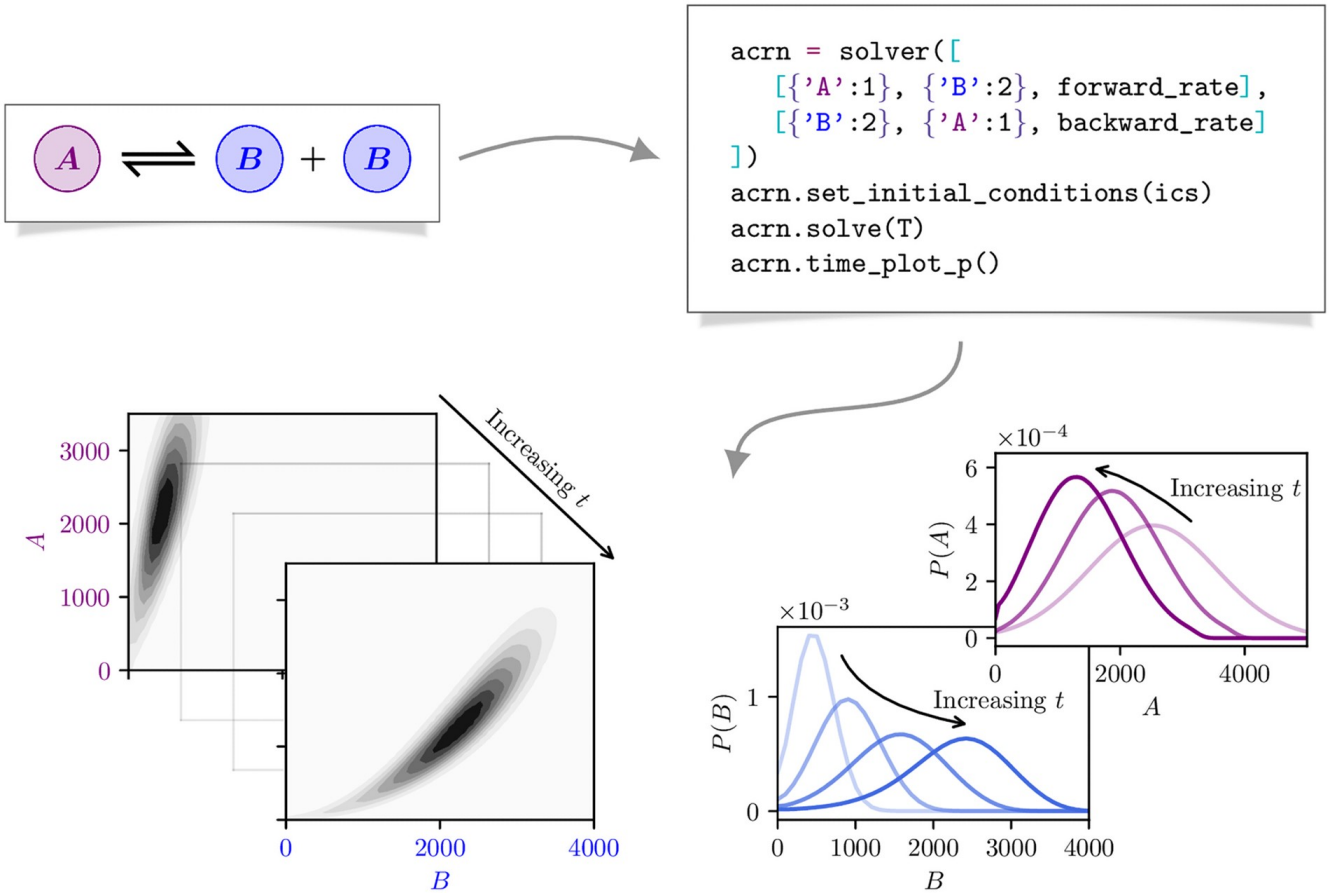
Schematic depiction of the Flips solver pipeline. Beginning with a (possibly augmented) chemical reaction network, we encode the reactions, set the initial distribution, and instruct the solver to determine the state distribution up to a terminal time *t* = *T* or terminal event. We may then plot the evolution of the state distribution, and its marginals, over time. For the sake of clarity, at this point we illustrate a classical chemical reaction network to highlight the simple one-to-one encoding of the reaction network, and the flavour of results the solver generates. The abstract and non-physical augmentation that distinguishes the ACME framework is demonstrated on more substantial networks in the Applications section.

First, the practitioner principally interested in simulation results with less focus on the numerical implementation details. With this in mind, the code is distributed as a package in the open-source and widely distributed Python programming language. The interface allows for a direct encoding of the reaction network structure without reference to the underling mathematical abstraction, as depicted in Fig 2.

Second, for the user interested in more control of the numerical implementation, the interface provides options to calibrate the state discretisation and tweak the flux limiter, as well as adjust the time stepping method and its order. Furthermore, the code has been written in a modular way that allows the more advanced user to retain the framework structure and implement a completely custom discretisation with minimal effort. For example, an implementation of a first-order finite difference scheme is included in the code, and achieved in little more than 20 lines of code. Building upon this, second- and third-order extensions are achieved in little more than 10 lines of code each.

We proceed to describe case studies in which we cast problems of interest from the literature in the ACME framework and solve them using the Flips software.

## Applications

Our aim is to combine a representation of highly non-trivial systems involving chemical reactions coupled to auxiliary processes to demonstrate how the hybrid structure of the ACME formulation of system (5) can be exploited to tackle an extensive class of problems. We benchmark the Flips solver by comparing simulations of each example problem with analytical results.

### First-passage times in self-regulated gene expression

Our first example is a model of regulated protein production as described in Friedman et al. [13] and Lin and Doering [40]. The reaction network may be written as

$$\begin{array}{c} \begin{array}{ccc} ⌀ & \overset{\;\mathrm{Hill}(\text{Protein})\;}{\to } & \text{mRNA,}\\ \text{mRNA} & \overset{\;\gamma B\;}{\to } & \text{mRNA}+\text{Protein,}\\ \text{mRNA} & \overset{\;\gamma \;}{\to } & ⌀\text{,}\\ \text{Protein} & \overset{\;\gamma_{0}\;}{\to } & ⌀\text{.} \end{array} \end{array}$$ (6)

The system comprises the transcription of mRNA molecules, the translation of protein molecules, and the degradation of both species. The dynamics are self-regulated because the mRNA transcription is regulated by the quantity of protein present, inducing a feedback loop. The notation Hill(⋅) represents that the feedback is modulated by a Hill function.

As described in Friedman et al. [13] and Lin and Doering [40], the characteristic timescale of the mRNA dynamics 1/*γ* is often exceeded by the protein lifetime timescale 1/*γ*_0_. In the limit as *γ*/*γ*_0_ → ∞, the mRNA acts instantaneously to produce a burst of proteins, and the reaction network reduces to

$$\begin{array}{c} \begin{array}{ccc} ⌀ & \overset{\;\mathrm{Hill}(\text{Protein})\;}{\to } & Z\cdot \text{Protein,}\\ \text{Protein} & \overset{\;\gamma_{0}\;}{\to } & ⌀\text{,} \end{array} \end{array}$$ (7)

where production in bursts is represented by the random variable *Z*, such that bursts occur at a rate Hill(Protein) with size distributed by *Z* ∼ Geo(1/*B*).

Thus we have two models from the literature describing a single genetic network. The finite mRNA lifetime model (6) is more general but less easy to analyse, while the infinitely fast mRNA model (7) is less general but simpler. Our aim is to use these two models as a first demonstration of how both descriptions (6) and (7) may be encapsulated and studied within the ACME framework of system (5). We then turn our attention to an insightful first-passage time problem studied for the second system (7). We describe how this problem too may be simulated and studied using the Flips solver, and significantly extend results previously reported.

Since the protein copy number is typically large, the proteins are taken on the continuum: represented by *x* where *d* = 1. Both the studies of Friedman et al. [13] and Lin and Doering [40] neglect the stochastic noise expressed by the diffusive term of order 1/Ω, and thus, for the sake of comparison, we too consider the noiseless limiting case as Ω → ∞.

In system (6), the mRNA will remain discrete, and the system may be written as

$$\begin{array}{c} \begin{array}{cc} \frac{\partial }{\partial t}p_{k}\left(x,t\right) & =-\frac{\partial }{\partial x}\left[\left(k\gamma B-\gamma_{0}x\right)p_{k}\left(x,t\right)\right]-\left(H\left(x\right)+\gamma k\right)p_{k}\left(x,t\right)\\ & \;+\gamma \left(k+1\right)p_{k+1}\left(x,t\right)+1_{k>0}H\left(x\right)p_{k-1}\left(x,t\right), \end{array} \end{array}$$ (8)

where $1_{k>0}$ denotes the indicator function, and *H* denotes the Hill function given by

$$\begin{array}{c} H\left(x\right)=r_{0}+r_{1}\frac{x^{n}}{1+x^{n}}. \end{array}$$ (9)

Each discrete state *k* represents the number of mRNA molecules present. In practice, we truncate the discrete mRNA state at some finite maximum 0 ≤ *k* ≤ *K*.

System (7) may be written as

$$\begin{array}{c} \frac{\partial }{\partial t}p\left(x,t\right)=\frac{\partial }{\partial x}\left[\gamma_{0}xp\left(x,t\right)\right]-H\left(x\right)p\left(x\right)+\int_{0}^{x}H\left(x-z\right)p\left(x-z,t\right)\frac{e^{-z/b}}{b}\;dz, \end{array}$$ (10)

for the mean continuum-scaled burst size *b* = *B*/Ω. The exponential burst kernel is a standard form for the burst-production jump density [13], and may be derived as the continuum scaling limit of the (discrete) geometrically distributed burst sizes [40].

Both reaction networks (6) and (7) fall within the ACME framework (5), and may then be solved numerically by application to the forms (8) and (10), respectively. To demonstrate the simplicity of using the Flips software, in Section 2 in S1 Appendix we present the code required to set up and simulate both reaction networks. Most of the code is simply a one-to-one translation of the discrete reaction network (each reaction is described by its reactants, products and rate). Intuitive instructions tell the solver to ignore diffusion, fix uniform initial conditions, solve over a specified time interval, and plot the distribution. This makes the software accessible to the end-user without the need to deal with the technicalities of the continuum approximation or its analysis. At the same time, the software allows fine-grained control of the discretisation of the state space and the differential operators, as well as the time stepping method and associated properties.

For the sake of comparing to results in Lin and Doering [40], we adopt the same parameter values, namely

$$\begin{array}{ccccccccccc} r_{0}=2, & & r_{1}=10, & & n=4, & & B=40, & & \gamma_{0}=1, & & \Omega =200. \end{array}$$ (11)

**Fig 3:**
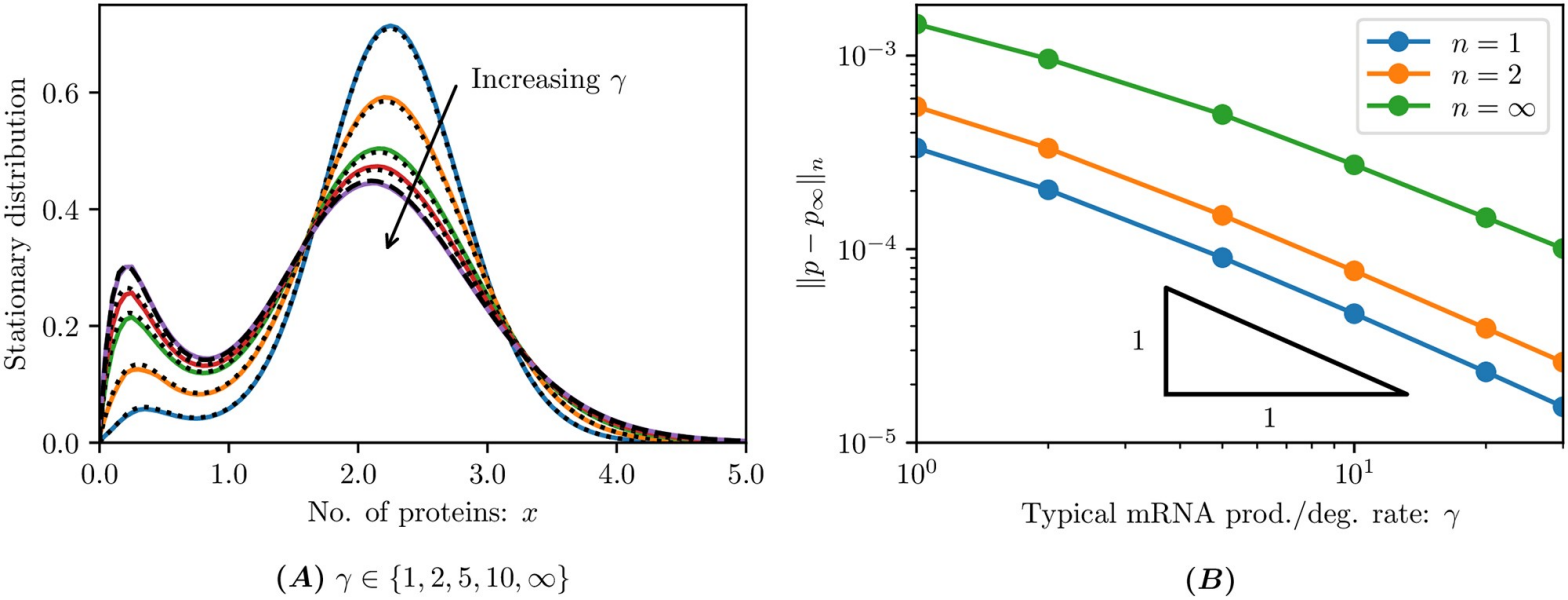
Comparison of self-regulated gene expression models. The stationary distributions of models (6) and (7) are compared while varying the typical mRNA production/degradation rate *γ*. **(A)** For finite *γ*, the solid curves show steady-state solutions of the continuum ACME form (8) compared to dotted curves which are the steady-state solution of the associated discrete CME system, retaining *K* = 30 discrete mRNA states in both cases. For the limiting case *γ* = ∞, the solid curve depicts the steady-state solution of the continuum ACME form (10) compared to the dashed curve which is the exact continuum solution *p*_∞_ in (12). The distributions are the marginal distributions of protein molecules, summing over the mRNA distribution. All continuum simulations used Δ*x* = 0.05, corresponding to lumping 10 discrete states together. **(B)** Convergence of the steady-state solution of the continuum ACME form (8) to the exact continuum solution *p*_∞_ in (12) in different *L*^*n*^ norms. The triangle shows the 1/*γ* convergence rate. We employed a discretisation of Δ*x* = 0.025, corresponding to lumping 5 discrete states together.

First, we explore the stationary distributions of these systems by comparing them to benchmark solutions. In the case of finite mRNA lifetime, the CME (with discrete state-space) associated with (6) is solved using the Flips solver to provide the stationary distribution of the discrete model. For infinitely fast-lived mRNA, the stationary distribution of the continuum (10), which we denote *p*_∞_(*x*), is given in Lin and Doering [40] by

$$\begin{array}{c} p_{\infty }\left(x\right)=\frac{c}{\gamma_{0}}e^{-x/b}x^{r_{0}/\gamma_{0}-1}{\left(1+x^{n}\right)}^{r_{1}/\left(n\gamma_{0}\right)}, \end{array}$$ (12)

where *r*_0_, *r*_1_ and *n* are the Hill function parameters, and *c* is a normalisation constant. These two distributions (black curves) are a useful comparison for the Flips solver (coloured curves) as illustrated in Fig 3.

In Fig 3A we plot the stationary distributions of (8) as given by the Flips solver (solid curves) and the full CME (dotted curves) for finite, increasing values of the mRNA production/degradation rate *γ*. The limiting case of *γ* = ∞ is plotted by simulation of (10) and compared to the exact continuum solution (dashed curve) given in (12). We see good agreement even though the Flips solver has lumped every ten protein molecules into a single discrete volume. Importantly, results in Lin and Doering [40] were obtained only in the limiting case of *γ*/*γ*_0_ → ∞, and thus intermediate values of *γ*/*γ*_0_ were beyond reach (see [40, Appendix F]). By retaining the finite lifetime of the mRNA molecules in formulation (8), the Flips software is able to match the CME over the entire range of relevant *γ*/*γ*_0_ values, and we observe the convergence of the finite mRNA lifetime scheme towards the infinitely fast-lived scheme. In Fig 3B we plot the norm of the discrepancy between the stationary distributions of these two schemes for increasing mRNA production/degradation rates *γ*. We observe a linear convergence of order 1/*γ*, which provides an error estimate not previously reported.

Having demonstrated the ACME framework’s utility in studying model accuracy by comparing stationary distributions, we proceed to study an important transient phenomenon. One striking feature of the regulated protein production is the bimodality of the stationary distribution: the nonlinear bursting supports both a low mode and a high mode where protein production and degradation are in balance. This stationary distribution is a dynamic equilibrium: in any single cell, the stochastic expression will be continually changing the number of protein molecules. The switching between these two modes can shed light on biological event timing. Choosing an arbitrary boundary of *x*_*c*_ = 0.825 proteins separating the high and low protein modes, we may ask: given any initial number of proteins *x*, how long will it take to cross the boundary *x* = *x*_*c*_, which we interpret to be a transition into the other mode. The mean switching time is given explicitly in Lin and Doering [40] by

$$\begin{array}{cc} T_{\text{low}\to \text{high}}\left(x\right) & =\frac{1-\gamma_{0}x_{c}V\left(x_{c}\right)e^{-M(x_{c})}}{H(x_{c})}+\int_{x_{c}}^{x}e^{-M(y)}V\left(y\right)\;dy, \end{array}$$ (13a)

$$\begin{array}{cc} T_{\text{high}\to \text{low}}\left(x\right) & =\int_{0}^{x}e^{-M(y)}[V(y)-V(\infty )]\;dy, \end{array}$$ (13b)

where

$$\begin{array}{ccc} M\left(x\right)=\log\frac{x}{H(x)}\;-\;\frac{x}{b}\;+\;\int_{0}^{x}\frac{H(y)}{\gamma_{0}y}\;dy, & & V\left(x\right)=-\;\int_{0}^{x}\;(\frac{1}{b\gamma_{0}y}\;+\;\frac{1}{\gamma_{0}yH\left(y\right)}\frac{dH}{dy})e^{M(y)}\;dy. \end{array}$$ (13c)

We would like to go beyond the mean switching time to study the full switching time distribution. Strategically leveraging the hybrid structure of the ACME framework, we may augment system (10) with an additional abstract state, so that the system is described by the densities *p*_0_ and *p*_1_. The original dynamics (10) are simulated on *p*_0_, in addition to a transition to the absorbing state *p*_1_ at a rate *α*, conditioned on whether the state has reached the opposite region. In other words, for an initial condition in the high-mode region *x* > *x*_*c*_, the absorption rate is $\alpha 1_{x<x_{c}}$, while for an initial condition in the low-mode region *x* < *x*_*c*_ the absorption rate is $\alpha 1_{x>x_{c}}$. In the limit as *α* → ∞, this models the opposite region as an immediately absorbing state. In practice, a sufficiently large value of *α* provides a good approximation. The switching time density is simply the probability mass that crosses the critical state *x*_*c*_ for the first time. With the solution of this augmented system, the switching time density is approximated by the instantaneous absorption rate.

**Fig 4:**
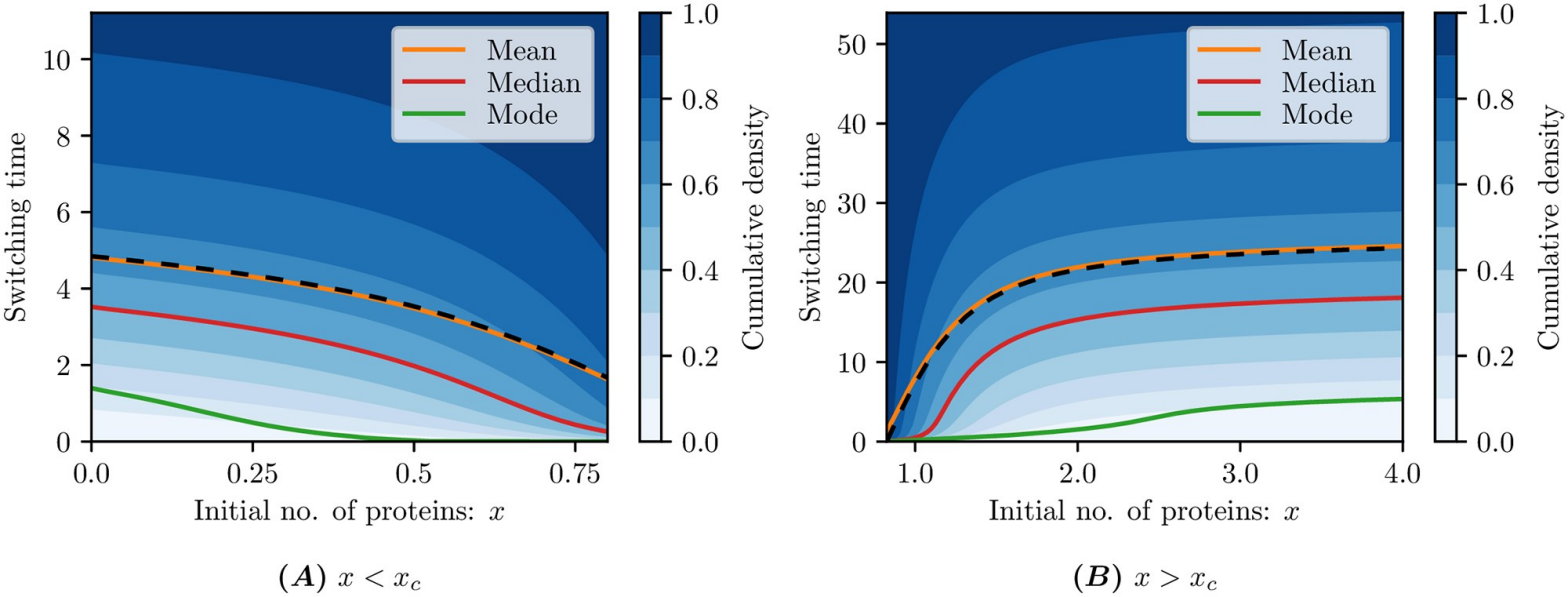
Switching time distributions. For different initial conditions *x* on either side of the critical *x*_*c*_ = 0.825 with an absorption rate *α* = 1000 and spatial discretisation of Δ*x* = 0.025 (i.e. lumping 5 states together) up to time *t* = 150. The exact mean switching time (13), is plotted as a black dashed curve.

In Fig 4 we plot the cumulative switching time density, along with the mean, median and mode. The exact solution (13) is plotted as a black dashed curve, with which the numerical solution closely agrees. Strikingly, the switching time distribution is highly skewed: for an initial number of proteins *x* ∈ (0.35, 2.25), the mean is at least an order of magnitude larger than the mode and is uniformly larger than the median. It is important for both the experimentalist and theoretician to keep this heavy tail in mind: with a small number of samples, switching times near the mode (and not the mean) are likely going to dominate the sample.

**Fig 5:**
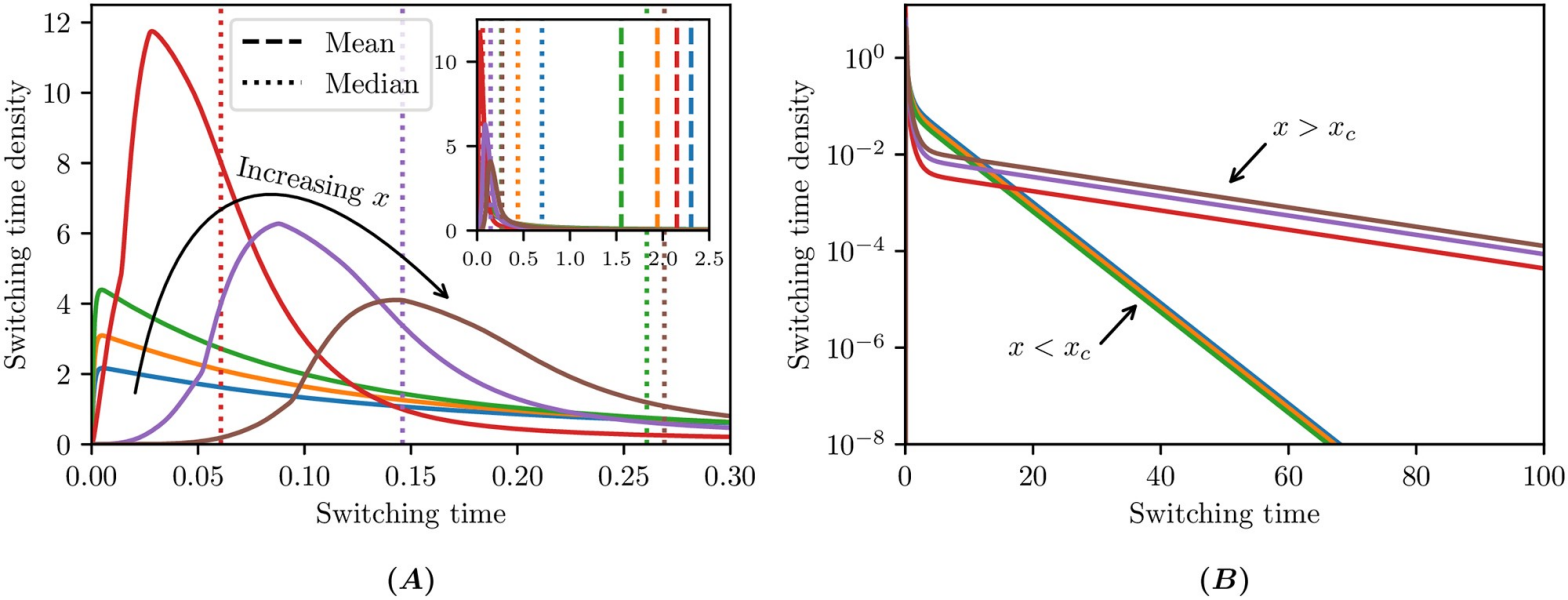
Extended switching time distributions. From the same data used to produce Fig 4 with the initial conditions *x* ∈ {0.7, 0.75, 0.8, 0.85, 0.9, 0.95}. **(A)** Zooming in to a small time interval resolves the modes, while **(A inset)** zooming out far enough to see the mean switching times shows the distribution skew. **(B)** The heavy tails are shown on a logarithmic scale for large times.

We plot a selection of the switching time densities in Fig 5, for initial conditions in both the low- and high-mode regions, to demonstrate just how far the mean is from the mode near the critical number of proteins *x*_*c*_. The heavy tails of these distributions are illustrated in Fig 5B, where we distinguish different large-time asymptotic behaviour for initial conditions below/above the critical boundary *x*_*c*_. The exponential decay in the switching time density suggests that the profile *p* converges to a separable solution of the form *p*(*x*, *t*) ∼ *T*(*t*)*Y*(*x*) for *T*(*t*) = e^−λ*t*^ motivating further analytical study.

In the models of self-regulated gene expression studied in this paper, the Flips solver is able to quantify model accuracy and convergence rates. By augmenting an abstract discrete state to capture a first passage, the Flips solver is able to produce the full first-passage time distribution, revealing a remarkably rich structure of heavy-tailed distributions, distinguished by the critical boundary between the high and low modes of protein expression. We proceed to study a population-level auxiliary process that cannot be captured by a master equation, showing that careful manipulation of physical constraints make the problem tractable within the ACME framework.

### Phenotypic selection

Many phenomena—such as changes to the ambient environment or the presence of media that affect metabolic or cell-cycle processes—can exert selection pressures. Importantly, such selection pressure may affect cells in a manner that depends on cell phenotype. For instance, recent studies provide an increasingly sharp understanding of how growth rates depend on gene expression [41]. This dependence motivates the development of models where an auxiliary process (capturing a selection pressure such as growth) is coupled to the single-cell gene expression kinetics. The ACME framework is capable of describing such population-level evolution, as we demonstrate in this second example.

We begin by considering the concentration of a particular protein that is heterogeneously distributed within a population due to stochastic gene expression (as in the previous case study). This is modeled as a birth–death process of a chemical species *X* within a single cell, governed by

$$\begin{array}{c} \begin{array}{ccccc} ⌀ & \overset{\;\lambda (x)\;}{\to } & \text{X} & \overset{\;\mu (x)\;}{\to } & ⌀\text{.} \end{array} \end{array}$$ (14)

The continuum approximation of process (14) takes the form [42]

$$\begin{array}{c} \frac{\partial }{\partial t}p\left(x,t\right)=-\frac{\partial }{\partial x}\left[\left(\lambda \left(x\right)-\mu \left(x\right)\right)p\left(x,t\right)\right]+\frac{1}{2\Omega }\frac{\partial^{2}}{\partial x^{2}}\left[\left(\lambda \left(x\right)+\mu \left(x\right)\right)p\left(x,t\right)\right]. \end{array}$$ (15)

We now seek to describe a growth process of a population of many such cells. We consider the protein concentration in daughter cells to be inherited from the mother cell. The growth *G* is to depend monotonically on protein concentration *x* (for example, when growth positively regulates cell-cycle stages and ultimately the rate of cell division), such that the population-level effect is preferential proliferation of cells with higher protein concentrations. This is modeled by adding a classical exponential growth term *G*(*x*)*p*(*x*, *t*) on the right-hand side of (15). Unfortunately, the resulting equation no longer conserves the total mass of *p*, therefore, *p* is no longer a probability distribution. This may be salvaged by normalisation [9, 10] where the total mass inflation due to growth is uniformly removed, yielding the governing equation

$$\begin{array}{c} \begin{array}{cc} \frac{\partial }{\partial t}p\left(x,t\right) & =-\frac{\partial }{\partial x}\left[\left(\lambda \left(x\right)-\mu \left(x\right)\right)p\left(x,t\right)\right]+\frac{1}{2\Omega }\frac{\partial^{2}}{\partial x^{2}}\left[\left(\lambda \left(x\right)+\mu \left(x\right)\right)p\left(x,t\right)\right]\\ & \;+G\left(x\right)p\left(x,t\right)-(\int_{0}^{\infty }G\left(z\right)p\left(z,t\right)\;dz)p\left(x,t\right). \end{array} \end{array}$$ (16)

By integrating over the state space *x*, the reader may verify that the total probability $\int_{0}^{\infty }p\left(x,t\right)\;dx$ is preserved in formulation (16).

It seems that only in the simplest of cases is equation (16) explicitly tractable. More worryingly, it seems that equation (16) is nonlinear and not of the general ACME form (5). We proceed to demonstrate how the nonlinear equation (16) is solved by a quantity satisfying an appropriately cast ACME equation of the generic form (5), thereby allowing us to use the Flips solver to tackle this population-level problem.

First, consider *q*, the solution of (16) without the nonlinear normalisation, namely

$$\begin{array}{c} \frac{\partial }{\partial t}q\left(x,t\right)=-\frac{\partial }{\partial x}\left[\left(\lambda \left(x\right)-\mu \left(x\right)\right)q\left(x,t\right)\right]+\frac{1}{2\Omega }\frac{\partial^{2}}{\partial x^{2}}\left[\left(\lambda \left(x\right)+\mu \left(x\right)\right)q\left(x,t\right)\right]+G\left(x\right)q\left(x,t\right). \end{array}$$ (17)

The normalised quantity $p=q/\int_{0}^{\infty }q\left(x,t\right)\;dx$ then satisfies the original Eq (16) and thus we have reduced the nonlinear problem (16) to the linear Eq (17).

Second, note that the linear Eq (17) may be cast in the general ACME form (5) by considering two discrete states (*k* = 0, 1): the first (*k* = 0) representing the density of the chemical species, and the second (*k* = 1) a phantom state to which a transition occurs from *k* = 0 with a propensity that is the negative of the growth rate, namely,

$$\begin{array}{cc} \frac{\partial }{\partial t}p_{0}\left(x,t\right) & =-\frac{\partial }{\partial x}\left[\left(\lambda \left(x\right)-\mu \left(x\right)\right)p_{0}\left(x,t\right)\right]+\frac{1}{2\Omega }\frac{\partial^{2}}{\partial x^{2}}\left[\left(\lambda \left(x\right)+\mu \left(x\right)\right)p_{0}\left(x,t\right)\right]\\ \; & \;-\left[-G\left(x\right)\right]p_{0}\left(x,t\right), \end{array}$$ (18)

$$\begin{array}{c} \frac{\partial }{\partial t}p_{1}\left(x,t\right)=\left[-G\left(x\right)\right]p_{0}\left(x,t\right). \end{array}$$ (19)

Ultimately, the ACME form of (18) and (19) allows us to use the Flips solver to tackle the nonlinear population-level Eq (16).

The quantity *p*_0_ is equivalent to *q*, except posed in a closed ACME form in (18) and (19), and thus is to be understood as a population density (as opposed to a probability density). The initial probability mass is to be distributed in *p*_0_, while *p*_1_(*x*, 0) = 0. The new state described by *p*_1_, is a phantom state, in that it does not represent a physical system configuration. To see this, consider, for example, the case of a positive growth rate *G*(*x*) > 0 then *p*_1_(*x*, *t*) ≤ 0 for all time *t*, and therefore *p*_1_ has no interpretation as a probability or population density. However, this non-physicality is of no concern: *p*_0_ solves (17), and we may safely ignore the value of the phantom state *p*_1_.

There are other ways of exploiting the observation that we may split the solution of (16) into a non-normalised form followed by a normalisation. For example, we could implement operator splitting by solving the non-normalised linear Eq (17) and adding a normalisation at each time step. The advantage of this approach would be that no additional discrete state need be introduced, and the numerical quantities remain O(1) without risk of numerical overflow at large times. When a smaller state space, or large-time simulations are crucial, this option should be considered. However, the principal advantage of our approach is that it does not require bespoke changes to the ACME framework or solver, and thus we pursue this approach for simplicity. Moreover, that the quantity *p*_0_ is a population density allows a direct quantification of the overall growth rate, as we now demonstrate.

For the purpose of numerical simulations, we take the underlying birth–death process studied in Lunz [42], namely

$$\begin{array}{ccc} \lambda (x)=\Lambda x(1-x), & & \mu (x)=x. \end{array}$$ (20)

We highlight that, for Λ > 1, there is a critical point *x*_*c*_ for which λ(*x*_*c*_) = *μ*(*x*_*c*_). On *x* ∈ (0, *x*_*c*_), birth dominates death λ(*x*) > *μ*(*x*), and the state increases towards *x*_*c*_, while for *x* ∈ (*x*_*c*_, 1), the opposite is true, and λ(*x*) < *μ*(*x*) and deaths dominate births driving the state down to *x*_*c*_. In the vicinity of the critical point stochastic effects become important [42].

**Fig 6:**
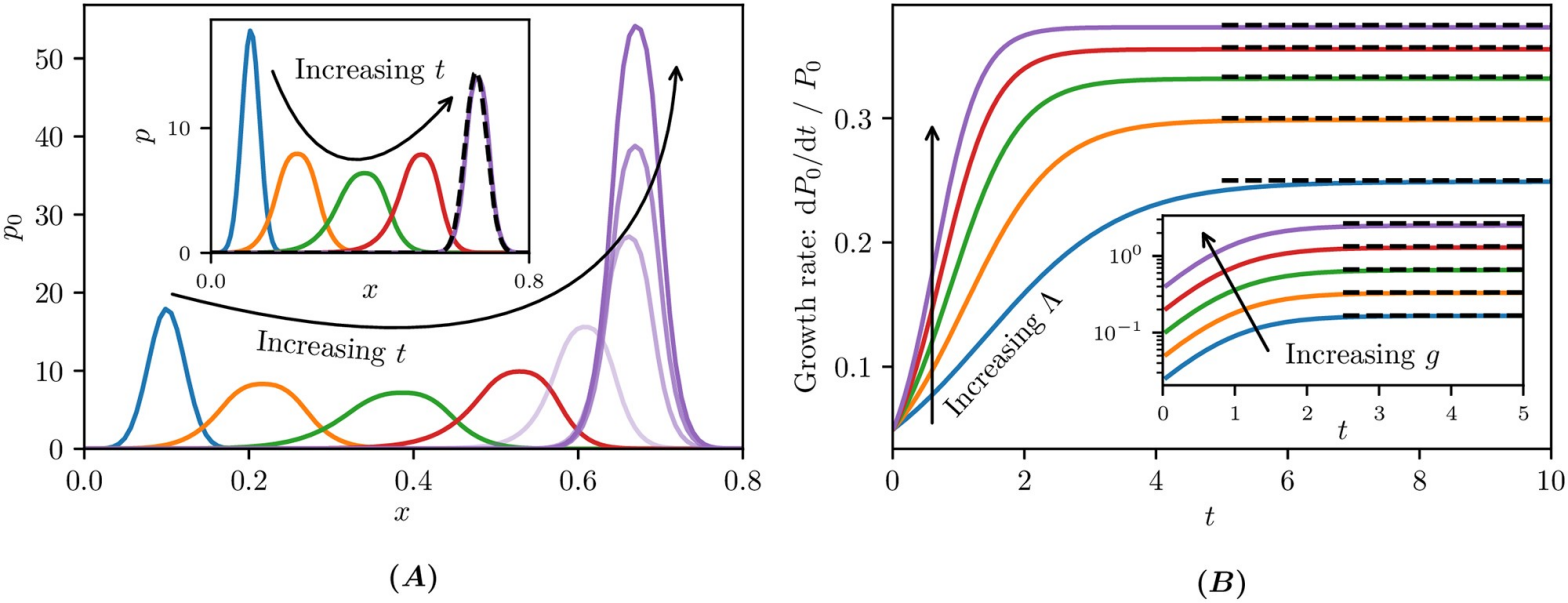
Chemical kinetics coupled to state-dependent growth. Simulations of (18) for *G*(*x*) = *gx* and the birth and death functions given in (20). Initial conditions are $p_{0}\left(x,0\right)=\exp\left(-10^{3}{\left(x-0.1\right)}^{2}\right)\sqrt{10^{3}/\pi }$ and *p*_1_(*x*, 0) = 0. **(A)** Density *p*_0_ for growth factor *g* = 0.5 and birth factor Λ = 3 at times *t* ∈ {0, 0.5, 1, 1.5, 2, 3, 4, 5}; **(A inset)** normalised density *p* = *p*_0_/*P*_0_ with *P*_0_ defined in (23) at times *t* ∈ {0, 0.5, 1, 1.5, 5}. The black dashed curve shows the asymptotic approximation of the limiting profile given by (S46) in Section 3 in S1 Appendix. **(B)** Growth rate (d*P*_0_/d*t*)/*P*_0_ for growth factor *g* = 0.5 and birth factor Λ ∈ {2, 2.5, 3, 3.5, 4}; **(B inset)** Λ = 3 and *g* ∈ {0.25, 0.5, 1, 2, 4}. The black dashed lines show the large time predictions ((S47) and (S50)). The state space discretisation was Δ*x* = 0.007 (i.e. lumping 7 states together).

In Fig 6A we show the solution of (18) with an initial Gaussian distribution centred at *x* = 0.1. The density *p*_0_(*x*, *t*) drifts toward higher values of *x* and from *t* ≈ 5 appears to have a stationary centre point after which time the growth is evident. In the inset of Fig 6A we plot the normalised density $p=p_{0}/\int_{0}^{\infty }p_{0}\left(x,t\right)\;dx$, and find that, for large times, the profile remains largely unchanged.

Motivated by the observation that the normalised density *p* appears to converge for large times *t*, we seek a separable solution of Eq (17). The calculations are detailed in Section 3 in S1 Appendix where we deduce that the profile tends to

$$\begin{array}{c} p_{0}\left(x,t\right)∼T\left(t\right)Y\left(x\right), \end{array}$$ (21a)

where

$$\begin{array}{ccc} T\left(t\right)=e^{r_{0}t}, & & Y\left(x\right)=\frac{1}{\sqrt{a\Omega \pi }}e^{-{\left(x-x_{c}\right)}^{2}/\left(a\Omega \right)}, \end{array}$$ (21b)

The limiting profile (21) is a Gaussian centred at *x* = *x*_*c*_ of width $O(1/\sqrt{a\Omega })$ growing at rate *r*_0_, which is given by

$$\begin{array}{c} r_{0}=G\left(x_{c}\right). \end{array}$$ (22)

For *G*(*x*) = *gx*, as used in our simulations, this becomes *r*_0_ = *g*(1 − 1/Λ).

These calculations serve as a useful gauge for our numerical simulations as illustrated in Fig 6. In the inset of Fig 6A we show the limiting normalised density (21), and observe good agreement with the numerical simulation at *t* = 5. We define the average growth rate of the numerical solution by (d*P*_0_/d*t*)/*P*_0_, where *P*_0_ denotes the total density in the *k* = 0 state, that is,

$$\begin{array}{c} P_{0}=\int_{0}^{\infty }p_{0}\left(x,t\right)\;dx. \end{array}$$ (23)

In Fig 6B we plot the average growth rate, and the predicted limiting growth rate (22) as black dashed lines, to which the numerical simulations converge for a range of system parameters.

To recap, the augmented reaction network considered in this section is not strictly physical, as we have incorporated negative propensities that induce negative “probabilities”. Nevertheless, by carefully crafting such a non-physical network, we were able to couple the single-cell reaction kinetics with an auxiliary growth process while taming the associated nonlinearity. In fact, the numerical simulations motivated the analytical study, which in turn uncovered insightful analytical information regarding the modes and long-time behaviour of the system, and allowed us to validate the simulations. We now turn our attention to an auxiliary fragmentation process that demonstrates the extensive coverage of the ACME framework.

### Divide and fragment

It can be crucially important to track the structure of a population, such as via the size, age, etc. of individual members. For example, in cell-cycle modeling, cell division may depend on cell size and cell-cycle stage [7, 43]. This motivates our final example, which does not stem from a classical chemical reaction network but a growth–fragmentation process, describing the evolution of a distribution of cells of different sizes subject to size increase (growth) and cell division (fragmentation). For an accessible introductory treatment, we refer the reader to Perthame [12, ch. 4]. In this class of models, we consider the independent state variable *x* to represents size, and we emphasise this distinction by denoting the number density *ρ*(*x*, *t*), with the volume density of cells of size *x* given by *xρ*(*x*, *t*). It is instructive to have in mind the concrete example of cell growth and division, and we will adopt the corresponding nomenclature, while keeping in mind that the models may be applied significantly more broadly both in biology [11] and beyond [14, 19].

The number density *ρ* is governed by the growth–fragmentation equation

$$\begin{array}{c} \frac{\partial }{\partial t}\rho \left(x,t\right)=-\frac{\partial }{\partial x}\left[g\left(x,t\right)\rho \left(x,t\right)\right]-B\left(x,t\right)\rho \left(x,t\right)+\int_{x}^{\infty }b\left(y,x,t\right)\rho \left(y,t\right)\;dy. \end{array}$$ (24)

The first term on the right-hand side of (24) represents the growth at a rate *g*(*x*, *t*). The final two terms represent the fragmentation. The first of these terms describes the reduction of cells of size *x* due to fragmentation, where *B*(*x*, *t*) is the rate of fragmentation of cells of size *x*. The integral term adds all the cells *y* > *x* that fragment into cells of size *x*, where *b*(*y*, *x*, *t*) is the fragmentation rate (per unit size) of cells of size *y* that fragment into a piece of size *x* and another piece of size *y* − *x*.

A no-flux boundary condition is imposed at *x* = 0, with the flux given by *g*(*x*, *t*)*ρ*(*x*, *t*), and a (sufficiently rapid) vanishing far-field condition as *x* → ∞. The model is typically studied in the absence of diffusion.

It is natural to expect the model to possess three physical features. First, the dynamics of the total number of cells is not to depend explicitly on the growth rate *g*, since growth is exclusively responsible for increasing cell size but not number. Second, the change of volume is not to depend explicitly on fragmentation, via *B* or *b*, since fragmentation is exclusively responsible for changing cell numbers but not total volume. Third, the rate of fragmentation of cells of size *y* into cells of size *x* is equal to the rate of fragmentation into cells of size *y* − *x* (for the case of binary fragmentation), since these events are equivalent. These features are guaranteed by ensuring that *B* and *b* satisfy certain constraints. Upon reflection, it is intuitive that *B* and *b* cannot be independent, as *B* quantifies the total fragmentation rate, while *b* represents the fragmentation rate (per unit size) into particular sizes. For the sake of completeness, in Section 4 in S1 Appendix we provide a comprehensive derivation of the algebraic conditions necessary to ensure physicality.

The salient point for our purposes is that the fragmentation can be guaranteed to respect the conservation properties described above by suitably relating *B* and *b*. In Section 4 in S1 Appendix we further show how the representation in equation (24) can be transformed to a rate and kernel, equivalent to the burst production process and conforming to the ACME structure of system (5). Thus, despite not being a chemical reaction network, the growth–fragmentation process is solvable with the Flips software.

We now seek a benchmark case with which we can compare numerical solutions. In Section 5 in S1 Appendix we combine results from Rooney et al. [44] and Cáceres et al. [45] to derive two closed-form solutions for two growth–fragmentation equations. In the first case, we use the time-invariant fragmentation kernel *b*(*y*, *x*, *t*) = 2*ay*^*k*−1^ for positive constants *a* and *k*, with no growth, that is, *g* ≡ 0. We may thus derive the form *B*(*x*, *t*) = *ax*^*k*^ (see Section 4 in S1 Appendix), whereupon the equation takes the form

$$\begin{array}{c} \frac{\partial }{\partial t}\rho \left(x,t\right)=-ax^{k}\rho \left(x,t\right)+\int_{x}^{\infty }2ay^{k-1}\rho \left(y,t\right)\;dy, \end{array}$$ (25)

and admits the self-similar solution

$$\begin{array}{c} \rho \left(x,t\right)=c{\left(at\right)}^{2/k}e^{-atx^{k}}, \end{array}$$ (26)

for an arbitrary constant *c*. A change of variables (see Section 5 in S1 Appendix) allows us to transform (26) to a solution of the equation

$$\begin{array}{c} \frac{\partial }{\partial t}\rho \left(x,t\right)=-\frac{\partial }{\partial x}[x\rho (x,t)]-\left(akx^{k}+1\right)\rho \left(x,t\right)+\int_{x}^{\infty }2aky^{k-1}\rho \left(y,t\right)\;dy, \end{array}$$ (27)

which is given by

$$\begin{array}{c} \rho \left(x,t\right)=c\;e^{-2t}{\left[a\left(e^{kt}-1\right)\right]}^{2/k}e^{-a\left(e^{kt}-1\right){\left(e^{-t}x\right)}^{k}}. \end{array}$$ (28)

Eq (27) describes growth linear in size, *g*(*x*, *t*) = *x*, and uniform decay.

Physically, the first example (25) describes a pure fragmentation process where the probability of a cell of size *y* being divided into cells of size *x* and *y* − *x* is uniform for all 0 ≤ *x* ≤ *y*. Thus, we expect the solution (26) to concentrate an increasing (and diverging) number density in a vicinity of the origin. Indeed, we see from (26) that, for any positive *x*, *ρ*(*x*, *t*) → 0 while *ρ*(0, *t*) → ∞ as *t* → ∞. The volume density, *xρ*(*x*, *t*), while eventually vanishing at every point *x* > 0, is conserved in total, as physically expected.

The second example (27) incorporates growth and decay, acting in competition with the fragmentation. How these competing forces balance in asymptotically large time is not trivial. We find from (28) that, in the limit as *t* → ∞, the density *ρ* converges to the profile

$$\begin{array}{c} \rho \left(x,t\right)∼ca^{2/k}e^{-ax^{k}}. \end{array}$$ (29)

We model Eq (25) by considering a species *X* undergoing a non-local fragmentation with kernel *b*(*y*, *x*) = *ay*^*k*−1^. This corresponds to a fragmentation rate of *ax*^*k*^ and a uniform fragmentation distribution (see Section 4 in S1 Appendix). Eq (27) requires two extra ingredients: production of *X* (in the sense of a reaction network, which captures growth in growth–fragmentation processes) and exponential transition to a new discrete state (decay). The species *X* is produced by a reaction at a rate proportional to its quantity

$$\begin{array}{c} ⌀\overset{\;X\;}{\to }X, \end{array}$$ (30)

in the diffusion-free limit Ω → ∞. Adding a discrete state allows us to incorporate the decay term, ultimately arriving at the system

$$\begin{array}{c} \frac{\partial }{\partial t}\rho_{0}\left(x,t\right)=-\frac{\partial }{\partial x}\left[x\rho_{0}\left(x,t\right)\right]+\int_{x}^{\infty }2ax^{k}\rho_{0}\left(y,t\right)\;dy-ax^{k}\rho_{0}\left(x,t\right)-\rho_{0}\left(x,t\right), \end{array}$$ (31)

$$\begin{array}{c} \frac{\partial }{\partial t}\rho_{1}\left(x,t\right)=\rho_{0}\left(x,t\right). \end{array}$$ (32)

For both examples, we use the initial condition given by the analytical solution at a nominal time *t* = *t*_0_, that is, *ρ*_0_(*x*, 0) = *ρ*(*x*, *t*_0_).

**Fig 7:**
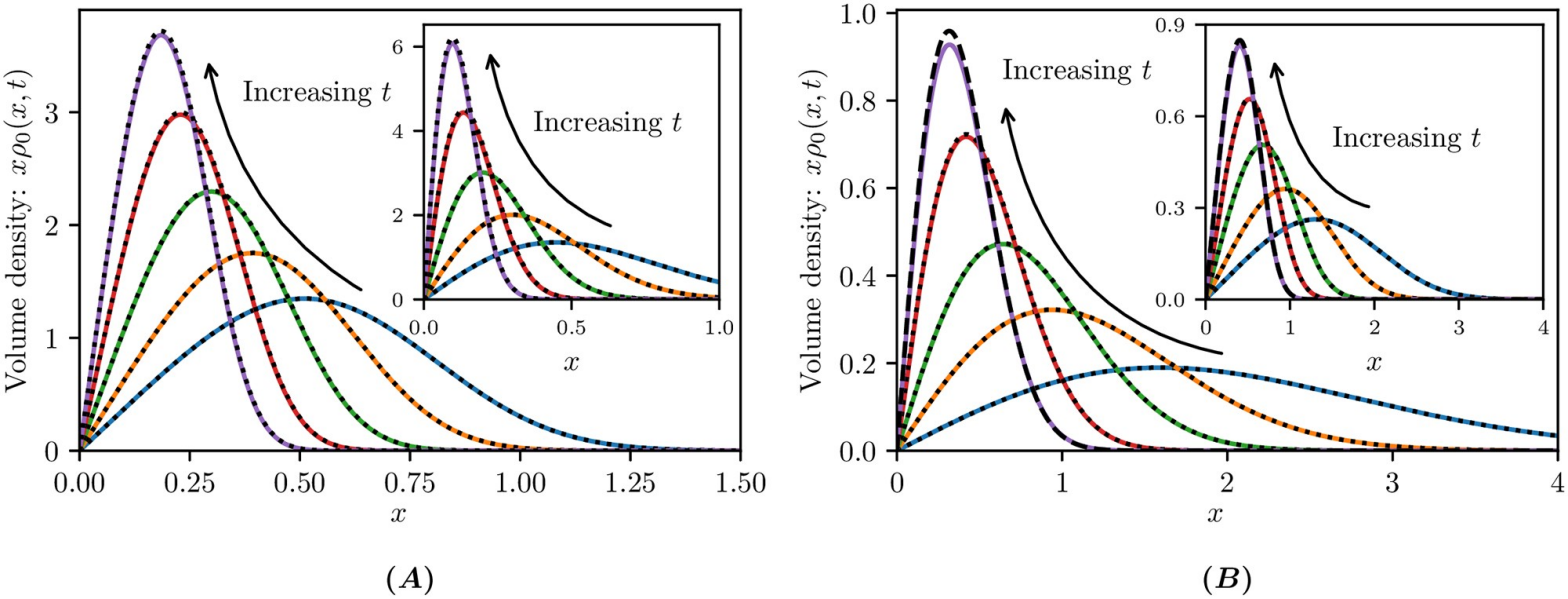
Growth–fragmentation processes. Analytical (black dotted curves) and numerical (coloured curves) solutions of growth–fragmentation equations at various times *t*. **(A)** Eq (25) with solution (26) at times *t* − *t*_0_ ∈ {0, 0.6, 2, 5, 10} where *t*_0_ = 0.5 and *k* = 3; **(A inset)** *k* = 2; **(B)** Eq (27) with solution (28) at times *t* − *t*_0_ ∈ {0.04, 0.12, 0.4, 2} where *t*_0_ = 0.02 and *k* = 2; **(B inset)** *t* − *t*_0_ ∈ {0.017, 0.07, 0.2, 2} where *t*_0_ = 0.01 and *k* = 3. The black dashed curves in (B) show the limiting profile as *t* → ∞ given by (29). Other parameters used are *a* = 5, Δ*x* = 0.005. Initial conditions are given by the analytical solution at time *t* = *t*_0_.

In Fig 7 we illustrate the analytical (black dotted curves) and numerical (coloured curves) solutions of Eqs (25) and (27) for *a* = 5 and *k* = 2, 3 at different times *t*. In Fig 7A, we simulate Eq (25) describing only fragmentation, which manifests as the volume density becoming concentrated around smaller sizes (near the origin). In contrast, Fig 7B shows simulations of Eq (27), where fragmentation acts in combination with decay to diminish the total volume density, and in competition with linear growth. The numerical solutions exhibit good agreement with the analytical solution. In Eq (27), the growth, decay, and fragmentation balance at asymptotically large time and the process converges toward the stationary distribution profile (29) as a black dashed curve in Fig 7B. We see that the long-time asymptotic behaviour is well approximated by the numerical solution already by time *t* = 2.

We have pointed out that both the dependent and independent quantities in the growth–fragmentation model (24) differ conceptually from their counterparts in the general reaction network model (5). Nonetheless, the model is of the same general form, and thus the solver is no less applicable. This is a powerful observation as it allows us to couple these two classes of models. As an example application, cell division has been reported to depend on gene expression pathways [46–48]. The ACME framework in tandem with the Flips software provides a generic framework in which such coupled models can be studied, alongside population-level effects and with view to transient dynamics (such as first-passage time problems).

## Discussion

The aim of this work is to establish a unifying framework for auxiliary processes coupled to arbitrary internal reaction kinetics that extend beyond classical chemical master equation descriptions. The Augmented Chemical Master Equation (ACME) framework that we introduce, is grounded in the classical CME. Considering the prohibitive challenge of solving the CME for large copy numbers, we take the Fokker–Planck continuum approximation for species that are typically large, while retaining discrete states for the remaining species. For the continuum states, we capture non-local effects, such as production in bursts.

We may leverage the discrete states to describe abstract system configurations that are not simply cardinal quantities, for example, binding/unbinding events, cell-fate decisions, and so forth. We demonstrate this use in modeling transient dynamics of self-regulated gene expression to solve the first-passage time problem. The method provides the complete first-passage time distribution of the bimodal system, extending previous results in the literature.

Another use for the discrete states is to describe non-physical phantom system configurations. The power of this approach is that it allows the underlying equations to capture dynamics beyond the reach of the classical chemical master equation. We demonstrate this by simulating population-level phenotypic selection. Typically, a nonlinear problem, we prove that the nonlinear component may be resolved separately by normalising the solution of a master equation with an exponential growth term, thereby reducing the problem to a linear one. The growth is captured by transition to a phantom state at a (non-physical) negative rate. The phantom state has no physical interpretation (with negative “probabilities”), but its presence makes the extended population-level dynamics tractable within the unified ACME framework, and thus directly solvable with the Flips solver.

Finally, the general problem tackled within the ACME framework is applicable to equations not typically associated with the chemical master equation, such as growth–fragmentation models. We demonstrate that the Flips solver provides an accurate tool for this class of models, and point to physical phenomena described by growth–fragmentation which are coupled to processes classically modeled with the master equation. This hints at further benefits of coupling the master equation to such auxiliary processes, motivating future work in this direction.

Our results were obtained by casting the diverse collection of examples in the ACME framework and solving these using the Flips solver. The run-time of each simulation depends strongly on the time and space discretisation. The time-step is typically dependent on both the space step as well as the specific problem structure and parameters (see (S29) in Section 1 in S1 Appendix). The examples presented here typically took between a couple of seconds and a couple of minutes on a single core of a standard laptop computer.

The first example of self-regulated gene expression was classical in the sense that the ACME framework describes a probability density of the state of a single cell, and there were no population-level effects. In the latter two examples of phenotypic selection and growth–fragmentation we observe that the density is not always positive nor is its integral conserved, whereby the ACME framework corresponds to the evolution of the expectation of an underlying stochastic process [7]. It is worth crystalising precisely when this does and does not occur. Crucially, system (5) is conservative if all kernels $B_{jk}$ are probability densities. This can be seen by integrating over the state space (using the zero-flux boundary conditions) to find that the total mass $\sum_{k\in K}\int_{R_{+}^{d}}p_{k}\left(x,t\right)\;dx$ is time invariant. We thus deduce that, when this kernel condition is satisfied, the total mass is conserved. Then, assuming an initial unit mass, it might seem that a probability distribution is retained. However, as is demonstrated in the example of phenotypic selection, if the rates are negative, we can end up with negative values of *p*_*k*_, which cannot be probability densities. The underlying cause is the negative discrete transition rates (even if *p*_*k*_ > 0 for all *k* and all time). The classical Markov jump process is defined via the probability of a transition during an infinitesimal interval d*t* (given by the rates times d*t*). This is where the probabilistic interpretation breaks down: a negative rate corresponds to a negative transition probability, thus no direct stochastic interpretation is possible.

While bursty production satisfies the aforementioned kernel condition, the growth–fragmentation equation does not (since the kernel B is two times a probability density, as explained in Section 4 in S1 Appendix, stemming from the fact that the division of a single cell leads to two cells). This results in number densities that are positive but not conserved. We conclude that both the non-local contributions and the extended use of negative rates are augmentations of the classical CME and Fokker–Planck framework, able to capture changes in the population composition due to auxiliary processes.

The current formulation admits some limitations. The ACME framework does not describe higher-order auxiliary processes such as aggregation, which may be of biological relevance. The reasons for this are manifold. While it is certainly possible to extend the solver to handle higher-order dynamics, a significant computational cost would accompany this high-order structure. From a theoretical perspective, the population density in the present formulation is so interpreted as it arises from an expectation of an underlying random variable (see, e.g., Supplementary Information 1 of Thomas [7]). The extension to nonlinear dynamics is nontrivial as the nonlinearity under expectation introduces covariances that are unaccounted for in the current theory.

Even though the continuum setting allows for significant dimensionality reduction by coarse graining, the framework (and hence the Flips solver) is subject to the curse of dimensionality. Machine-learning-based approaches have enormous potential to make high dimensional systems tractable [49] and escape this limitation.

The examples illustrated in this work are intended to demonstrate some of the basic building blocks of the framework and solver. In this spirit, tractable examples were chosen to compare with previous work and analytical calculations. Nevertheless, we stress that more elaborate and sophisticated models may be tackled with this framework. We confined ourselves to no more than one auxiliary process in each example, however, this is not a fundamental limitation, and several such processes may be combined.

The hybrid network structure combining the continuum and discrete descriptions, alongside non-local dynamics, applied in versatile, often non-physical, ways, such as the use of negative reaction rates, significantly extends the scope of the classical master equation formulation. Our hope is that this augmented approach, and the accompanying software, puts a richer class of models and complex coupled processes firmly within reach.

## Supporting information

S1 Appendix Supplementary numerical, analytical, and computational descriptions. Section 1 includes details of the numerical scheme and its analysis. Section 2 provides code examples for the self-regulated genetic expression models described in this paper. In Section 3, an asymptotic analysis is performed for the selection model presented earlier. Section 4 introduces properties of the growth–fragmentation models studied here, including discretisation details. Section 5 develops two explicit solutions of growth–fragmentation equations to serve as benchmarks.
(PDF)
