# Supplementary material for: Beyond the chemical master equation: Stochastic chemical kinetics coupled with auxiliary processes
Source: PLoS Comput Biol. 2021 Jul 28;17(7):e1009214. doi: 10.1371/journal.pcbi.1009214 (PMC8352075; doi:10.1371/journal.pcbi.1009214)
Supplement: S1 Appendix — Section 1 includes details of the numerical scheme and its analysis. Section 2 provides code examples for the self-regulated genetic expression models described in this paper. In Section 3, an asymptotic analysis is performed for the selection model presented earlier. Section 4 introduces properties of the growth–fragmentation models studied here, including discretisation details. Section 5 develops two explicit solutions of growth–fragmentation equations to serve as benchmarks. (PDF) [file pcbi.1009214.s001.pdf]

Supplementary Information

Beyond the chemical master equation: stochastic chemical kinetics coupled with auxiliary processes

Davin Lunz, Gregory Batt, Jakob Ruess, J. Frédéric Bonnans

S1 Appendix

Equation numbering in the Supplementary Information is preceded by an 'S'. Labels without a preceding 'S' are references to the main paper.

1 Numerical solver

In this appendix, we detail the numerical scheme employed to solve system (5) from the main paper, and we prove a result necessary for its stability.

We begin by introducing relevant notation. The fixed state scale is denoted by Δx, whereby the uniform grid is $\{0, N_1\Delta x\} \times \cdots \times \{0, N_d\Delta x\}$. We index the numerical scheme by integer vectors, where $\boldsymbol{j}\Delta x = (j_1\Delta x, \ldots, j_d\Delta x)$, and write the discrete probability mass as $(p_k)^n_{\boldsymbol{j}} = p_k(\boldsymbol{j}\Delta x, t^n)$, for a sequence of time steps $\{t^1, \ldots, t^n\}$. We similarly use the $\boldsymbol{j}$ subscript to denote evaluation of any function at the point $\boldsymbol{x} = \boldsymbol{j}\Delta x$.

The discrete transitions are added to the explicit scheme in the classical manner. By summing over $k \in \mathcal{K}$ in (5), one finds that the discrete transitions conserve probability mass. The first-order differential operator describing the drift is one of the trickiest parts of the numerical implementation. We proceed to describe the finite-volume scheme used to discretise the differential operator in Section 1.1, followed by the discretisation of the integral operator for a burst kernel in Section 1.2, and finish by deriving a condition on the time step for scheme stability in Section 1.3.

1.1 Finite volume scheme

We employ the second-order, central, flux-limited finite-volume scheme introduced in Kurganov and Tadmor [1] applied for each reaction $i \in \mathcal{I}$ one-dimensionally in the $\boldsymbol{e}_i$ direction. For the sake of concreteness, we demonstrate the scheme for a system comprising a single reaction in the direction $\boldsymbol{e}$ with rate r and no non-local effects or additional discrete states, which we write as

$$\frac{\partial}{\partial t}p(\boldsymbol{x}, t) = -\frac{\partial}{\partial \boldsymbol{e}}\left[r(\boldsymbol{x}, t)p(\boldsymbol{x}, t)\right] + \frac{1}{2\Omega}\frac{\partial^2}{\partial \boldsymbol{e}^2}\left[r(\boldsymbol{x}, t)p(\boldsymbol{x}, t)\right]. \tag{S1}$$

The directional derivative highlights the one-dimensional nature of the differential terms. We write the scheme in a semi-discrete manner: we discretise the state operators while leaving the time derivative on the left-hand side to be dealt with by a separate time-marching scheme, namely an explicit Runge–Kutta method (or multi-level method) as discussed in Kurganov and Tadmor [1]. The scheme has the form

$$\begin{aligned}\frac{\partial}{\partial t}p_{\boldsymbol{j}}(t) = &-\frac{1}{\Delta x}\left[r_{\boldsymbol{j}}(t)\left(p_{\boldsymbol{j}}(t) + (\nabla_{\boldsymbol{e}}p)_{\boldsymbol{j}}(t)\frac{\Delta x}{2}\right) - r_{\boldsymbol{j}-\boldsymbol{e}}(t)\left(p_{\boldsymbol{j}-\boldsymbol{e}}(t) + (\nabla_{\boldsymbol{e}}p)_{\boldsymbol{j}-\boldsymbol{e}}(t)\frac{\Delta x}{2}\right)\right] \\ &+ \frac{1}{2\Omega(\Delta x)^2}\left[r_{\boldsymbol{j}}(t)p_{\boldsymbol{j}+e}(t) - 2r_{\boldsymbol{j}}(t)p_{\boldsymbol{j}}(t) + r_{\boldsymbol{j}}(t)p_{\boldsymbol{j}-\boldsymbol{e}}\right],\end{aligned} \tag{S2a}$$

where the flux limiting modulates the gradient discretisation via

$$(\nabla_{\boldsymbol{e}} p)_{\boldsymbol{j}} = \operatorname{minmod}\left(\theta \frac{p_{\boldsymbol{j}} - p_{\boldsymbol{j}-\boldsymbol{e}}}{\Delta x}, \frac{p_{\boldsymbol{j}+\boldsymbol{e}} - p_{\boldsymbol{j}-\boldsymbol{e}}}{2\Delta x}, \theta \frac{p_{\boldsymbol{j}+\boldsymbol{e}} - p_{\boldsymbol{j}}}{\Delta x}\right), \tag{S2b}$$

for

$$\operatorname{minmod}(a, b) = \frac{1}{2}(\operatorname{sgn}(a) + \operatorname{sgn}(b)) \min(|a|, |b|), \tag{S2c}$$

$$\operatorname{minmod}(a, b, c) = \operatorname{minmod}(\operatorname{minmod}(a, b), c). \tag{S2d}$$

The flux limiting ensures no spurious oscillations are introduced into the numerical solution. The parameter $\theta \in [1, 2]$ influences the nature of the flux limiter: interpolating between a less oscillatory scheme ($\theta = 1$) and a less dissipative scheme ($\theta = 2$). In all the examples presented here we fix $\theta = 1$, but refer the reader to Kurganov and Tadmor [1] and references therein for further discussion. Zero normal flux at the boundaries (for each reaction) ensures conservation, and is achieved by neglecting flux terms whose support would extend beyond the grid.

For the conservation equations studied in Kurganov and Tadmor [1], a sufficiently small time step Δt^n obeying the Courant–Friedrichs–Lewy (CFL) condition derived in Kurganov and Tadmor [1], the scheme is monotone and satisfies a maximum principle whereby the ℓ^∞-norm of the discrete values is non-increasing (see Theorem 5.1, Corollaries 5.1 and 5.2 in Kurganov and Tadmor [1]).

The setting of conservation equations is more restrictive than our model (5), since we incorporate diffusion, non-local effects, discrete transitions, as well as state-dependent flux functions. Therefore, the associated CFL condition is not directly applicable. Moreover, we cannot expect to obtain a maximum principle, since the continuum dynamics do not respect a maximum principle: consider, for example, any reaction network whose stationary distribution is not uniform, and take initial conditions more spread than the stationary distribution. Nonetheless, the proof of Theorem 5.1 in Kurganov and Tadmor [1] is adapted in Section 1.3 to account for these additional contributions to provide a monotonicity condition: for a small enough time step Δt^n artificial oscillations will not be introduced into the numerical solution.

1.2 Discretising the burst operator

The non-local contributions on the second line of (5) are made up of probability mass lost to non-local effects, and the integral term describing mass gained due to these effects emanating from other states and coming into $\boldsymbol{x}$. In this section, we describe the discretisation for the class of non-local kernels describing production in bursts. A discussion on the discretisation of fragmentation kernels appears in Section 4, which builds on the present analysis.

To simplify the notation, we consider a single bursting process, and thus remove the subscripts j and k, taking

$$\frac{\partial}{\partial t} p(\boldsymbol{x}, t) = -f(\boldsymbol{x}, t) p(\boldsymbol{x}, t) + \|\boldsymbol{e}\| \int_{\boldsymbol{x} - z\boldsymbol{e} \in \mathbb{R}_+^d} f(\boldsymbol{x} - z\boldsymbol{e}, t) p(\boldsymbol{x} - z\boldsymbol{e}, t) \mathcal{B}(z\|\boldsymbol{e}\|) \, \mathrm{d}z. \tag{S3}$$

Burst kernels take the form

$$\mathcal{B}(y) = \begin{cases} \dfrac{\mathrm{e}^{-y/b}}{b}, & y > 0, \\ 0, & y < 0, \end{cases} \tag{S4}$$

where b is the mean burst size, and the burst kernel has an unbounded support. On the truncated state space, we respect the finite boundary by retaining only those bursts that

transfer probability mass between two states within the finite domain. This amounts to changing the domain of integration to $\boldsymbol{x} - z\boldsymbol{e} \in [0, N_1\Delta x] \times \cdots \times [0, N_d\Delta x]$. To ensure conservation, we must account for the corresponding reduction in mass loss. This is achieved in our scheme by focusing on the bursts emanating *from* each $\boldsymbol{x}$ (as opposed to the integral representation in (S3), which describes the bursts *into* the state $\boldsymbol{x}$). For each $\boldsymbol{j}\Delta x$ on the grid, we find the largest burst that remains within the domain:

$$m_{\max} = \max_m \{m \mid \boldsymbol{j} + m\boldsymbol{e} \in [0, N_1] \times \cdots \times [0, N_d]\}. \tag{S5}$$

The scheme ensures that mass is lost and gained equally. The losses

$$\frac{\partial}{\partial t} p_{\boldsymbol{j}}(t) = \sum_{m=1}^{m_{\max}} -f_{\boldsymbol{j}}(t)\mathbb{P}_m + (\text{other contributions}), \tag{S6}$$

are balanced by the gains: for all $1 \leq m \leq m_{\max}$

$$\frac{\partial}{\partial t} p_{\boldsymbol{j}+m\boldsymbol{e}}(t) = f_{\boldsymbol{j}}(t)\mathbb{P}_m + (\text{other contributions}), \tag{S7}$$

where we have denoted the probability of a jump of $m\boldsymbol{e}$ grid points by $\mathbb{P}_m$.

To determine the jump probability $\mathbb{P}_m$, we recall that in the finite-volume framework, the value at a point represents the probability mass of a cell around at that point. Retaining a one-dimensional projection,

$$p_{\boldsymbol{j}}(t) \approx \int_{-\Delta x\|\boldsymbol{e}\|/2}^{\Delta x\|\boldsymbol{e}\|/2} p(\boldsymbol{j}\Delta x + y\boldsymbol{e}/\|\boldsymbol{e}\|, t)\, \mathrm{d}y. \tag{S8}$$

The probability to burst into a cell $m\boldsymbol{e}$ units away, is given by

$$\mathbb{P}_m = \frac{1}{\Delta x\|\boldsymbol{e}\|} \int_{-\Delta x\|\boldsymbol{e}\|/2}^{\Delta x\|\boldsymbol{e}\|/2} \int_{(m-1/2)\Delta x\|\boldsymbol{e}\|}^{(m+1/2)\Delta x\|\boldsymbol{e}\|} \mathcal{B}(y)\, \mathrm{d}y\, \mathrm{d}x, \tag{S9}$$

where we assume a uniform distribution in the cell of origin. For jump kernels of the form (S4), this probability may be calculated analytically, namely

$$\mathbb{P}_m = \frac{b}{\Delta x\|\boldsymbol{e}\|} q^{m-1}(1-q)^2, \qquad \text{where} \qquad q := \mathrm{e}^{-\Delta x\|\boldsymbol{e}\|/b}. \tag{S10}$$

This discretisation preserves the mean burst size (on an infinite domain). To see this, note that the mean burst size may be calculated via

$$\mathbb{E}[\text{jump size}] = \Delta x\|\boldsymbol{e}\| \sum_{m=1}^{\infty} m\mathbb{P}_m = b\frac{(1-q)^2}{q} \sum_{m=1}^{\infty} mq^m. \tag{S11}$$

Since $q < 1$, we may evaluate the series via

$$\sum_{m=1}^{\infty} mq^m = q\sum_{m=1}^{\infty} mq^{m-1} = q\frac{\mathrm{d}}{\mathrm{d}q}\sum_{m=1}^{\infty} q^m = q\frac{\mathrm{d}}{\mathrm{d}q}\left(\frac{1}{1-q}\right) = \frac{q}{(1-q)^2}. \tag{S12}$$

From (S11) and (S12) it follows that

$$\mathbb{E}[\text{jump size}] = b. \tag{S13}$$

1.3 Monotonicity condition

In this appendix, we outline the proof in Kurganov and Tadmor [1] that the numerical scheme satisfies a maximum principle, and show how the argument may be adapted to system (5). The equation under consideration is

$$\frac{\partial}{\partial t}p(x,t) = -\frac{\partial}{\partial x}[F(p(x,t))]. \tag{S14}$$

where $x \in \mathbb{R}$ is a scalar. We denote the values of the numerical scheme at time step n by $p_{\boldsymbol{j}}^n$, where $\boldsymbol{j}$ indexes the finite volume. The CFL condition is

$$\frac{\Delta t^n}{\Delta x}\|F'\|_\infty \leq \frac{1}{8}, \tag{S15}$$

where Δt^n denotes the size of the nth time step, and Δx denotes the step size in state space. From Theorem 5.1 in Kurganov and Tadmor [1] it holds that, if the CFL condition (S15) is satisfied, then the ℓ^∞-norm of the scheme is non-increasing with a first-order Euler time step, that is,

$$\max_{\boldsymbol{j}} p_{\boldsymbol{j}}^{n+1} \leq \max_{\boldsymbol{j}} p_{\boldsymbol{j}}^n. \tag{S16}$$

Corollaries 5.1 and 5.2 in Kurganov and Tadmor [1] extend this result to higher-order time-stepping methods.

Our first observation is that, in preserving a uniform grid, the advection and diffusion operators may be projected onto one dimension, while the CFL condition (S15) was derived for two-dimensional advection. Restriction to one dimension allows us to relax the CFL condition (S15) by a factor of 2. To understand this, we highlight that the proof expresses the explicit Euler time step as

$$p_{\boldsymbol{j}}^{n+1} = p_{\boldsymbol{j}}^n + \Delta t^n \left\{ \sum_{i=1}^{d} \sum_{k=1}^{4} \alpha_{i,k} C_{i,k}(\{p_{\boldsymbol{j}}^n\}_{\boldsymbol{j}}) \right\}, \tag{S17}$$

where $C_{i,k}$ are functions satisfying a local maximum principle. The discretisation admits a decomposition of the $p_{\boldsymbol{j}}^n$ contribution of the form

$$p_{\boldsymbol{j}}^n = \sum_{i=1}^{d} \frac{C_{i,3}(\{p_{\boldsymbol{j}}^n\}_{\boldsymbol{j}}) + C_{i,4}(\{p_{\boldsymbol{j}}^n\}_{\boldsymbol{j}})}{2d}, \tag{S18}$$

which allows the right-hand side of (S17) to be expressed as a linear combination of $C_{i,k}$ terms. The goal is to prove that the right-hand side may be expressed as a *convex* combination of $C_{i,k}$ terms, that is, where the coefficient of each $C_{i,k}$ term is positive and the sum of all coefficients is one. It may be seen from the formulae for the coefficients shown in Kurganov and Tadmor [1] that they sum to one, and we focus on demonstrating their positivity. For each dimension i, it may be shown that $\alpha_{i,1}$ and $\alpha_{i,2}$ are non-negative by construction of the scheme, while the other two terms are bounded by

$$|\alpha_{i,k}| \leq \frac{2\|F'\|_\infty}{\Delta x}, \qquad \text{for} \qquad k = 3, 4. \tag{S19}$$

With no control over the sign of these $\alpha_{i,k}$ terms for $k = 3, 4$, we may guarantee that the coefficients remain positive by choosing Δt^n small enough to ensure that the $p_{\boldsymbol{j}}^n$ contribution dominates the (possibly) negative contribution via

$$2\frac{\Delta t^n}{\Delta x}\|F'\|_\infty \leq \frac{1}{2d}. \tag{S20}$$

From (S20) we see that the original CFL condition (S15) from Kurganov and Tadmor [1] may be relaxed by a factor of 2, even when system (5) is multidimensional, since the differential operators act on a single dimension, that is, d is effectively one in our discretisation.

We may extend the equation under consideration to incorporate the time- and state-varying fluxes, discrete states, and non-local effects described in system (5). In this general case, we cannot hope to obtain a maximum principle since the flux function variations in state can act to concentrate probability mass, while discrete transition rates may be negative resulting in exponential growth (as in the phenotypic selection case study). Therefore, the continuous dynamics do not respect a maximum principle. Instead, we focus on monotonicity. A scheme is called monotone if it may be expressed in the form

$$p_{\boldsymbol{j}}^{n+1} = \sum_{\boldsymbol{j}} \alpha_{\boldsymbol{j}}^n p_{\boldsymbol{j}}^n, \qquad \text{for} \qquad \alpha_{\boldsymbol{j}}^n \geq 0. \tag{S21}$$

Artificial oscillations driving numerical instability may result from insufficiently small time steps which violate scheme monotonicity. In analogy with the previous argument, our aim is to express the explicit Euler scheme in a form akin to (S17), and find a time step Δt^n small enough to guarantee that the coefficients of $C_{i,k}$ remain positive. This suffices to ensure monotonicity since the local maximum principle satisfied by the $C_{i,k}$ terms guarantees that these terms may be expressed as convex combinations of local $p_{\boldsymbol{j}}^n$ terms.

Neglecting terms that are inhomogeneous in the discretised system (such as the integral terms whose non-local contributions come from states of index $\hat{j} \neq j$ and sources from discrete states of index $\hat{k} \neq k$), we rewrite (5) as

$$\frac{\partial}{\partial t} p_k(\boldsymbol{x}, t) = -h_k(\boldsymbol{x}, t) p_k(\boldsymbol{x}, t) + \sum_{i \in \mathcal{I}} -\frac{\partial}{\partial \boldsymbol{e}_i} \left[r_{ik}(\boldsymbol{x}, t) p_k(\boldsymbol{x}, t) \right] + \frac{1}{2\Omega} \frac{\partial^2}{\partial \boldsymbol{e}_i^2} \left[r_{ik}(\boldsymbol{x}, t) p_k(\boldsymbol{x}, t) \right] + (\text{source terms}), \tag{S22}$$

where we write the differential operators using directional derivatives to emphasise their one-dimensional nature, and the rate h_k encapsulates the homogeneous contributions of discrete transitions and non-local effects, namely

$$h_k(\boldsymbol{x}, t) = \sum_{j \in \mathcal{J}} f_{jk}(\boldsymbol{x}, t) + \sum_{\ell \in \mathcal{K}} g_{k\ell}(\boldsymbol{x}, t). \tag{S23}$$

Focusing on a single value of k, we henceforth drop the subscript (but recycle the index k for separate use). The explicit Euler scheme may be written as

$$p_{\boldsymbol{j}}^{n+1} = p_{\boldsymbol{j}}^n + \Delta t^n \left\{ -h_{\boldsymbol{j}}^n p_{\boldsymbol{j}}^n + \sum_{i \in \mathcal{I}} \left[\frac{1}{2\Omega(\Delta x)^2} \left[(r_i p)_{\boldsymbol{j}+\boldsymbol{e}_i}^n - 2(r_i p)_{\boldsymbol{j}}^n + (r_i p)_{\boldsymbol{j}-\boldsymbol{e}_i}^n \right] + \sum_{k=1}^{4} \alpha_{i,k} C_{i,k}(\{p_{\boldsymbol{j}}^n\}_{\boldsymbol{j}}) \right] \right\} + (\text{source terms}). \tag{S24}$$

The coefficients $\alpha_{i,1}$ and $\alpha_{i,2}$ are non-negative, while the remaining coefficients are bounded (see (S19)) via

$$|\alpha_{i,k}| \leq \frac{2\|r_i\|_\infty}{\Delta x}, \qquad \text{for} \qquad k = 3, 4. \tag{S25}$$

Collecting terms of $p_{\boldsymbol{j}}^n$, we see that

$$p_{\boldsymbol{j}}^{n+1} = p_{\boldsymbol{j}}^n \left(1 - \Delta t^n h_{\boldsymbol{j}}^n - \sum_{i\in\mathcal{I}} \frac{\Delta t^n (r_i)_{\boldsymbol{j}}^n}{\Omega(\Delta x)^2}\right) + \Delta t^n \sum_{i\in\mathcal{I}} \left(\alpha_{i,3} C_{i,3}(\{p_{\boldsymbol{j}}^n\}_{\boldsymbol{j}}) + \alpha_{i,4} C_{i,4}(\{p_{\boldsymbol{j}}^n\}_{\boldsymbol{j}})\right)$$
$$+ \sum_{\boldsymbol{j}} \beta_{\boldsymbol{j}} p_{\boldsymbol{j}}^n + (\text{source terms}), \tag{S26}$$

for non-negative constants $\beta_{\boldsymbol{j}} \geq 0$. Decomposing $p_{\boldsymbol{j}}^n$ in analogy to (S18), via

$$p_{\boldsymbol{j}}^n = \sum_{i\in\mathcal{I}} \frac{C_{i,3}(\{p_{\boldsymbol{j}}^n\}_{\boldsymbol{j}}) + C_{i,4}(\{p_{\boldsymbol{j}}^n\}_{\boldsymbol{j}})}{2|\mathcal{I}|}, \tag{S27}$$

we may write

$$p_{\boldsymbol{j}}^{n+1} = \sum_{\boldsymbol{j}} \beta_{\boldsymbol{j}} p_{\boldsymbol{j}}^n + (\text{source terms})$$
$$+ \sum_{i\in\mathcal{I}} \left[\frac{1}{2|\mathcal{I}|}\left(1 - \Delta t^n h_{\boldsymbol{j}}^n - \sum_{i\in\mathcal{I}} \frac{\Delta t^n (r_i)_{\boldsymbol{j}}^n}{\Omega(\Delta x)^2}\right) + \Delta t^n \alpha_{i,3}\right] C_{i,3}(\{p_{\boldsymbol{j}}^n\}_{\boldsymbol{j}}) + \tag{S28}$$
$$\left[\frac{1}{2|\mathcal{I}|}\left(1 - \Delta t^n h_{\boldsymbol{j}}^n - \sum_{i\in\mathcal{I}} \frac{\Delta t^n (r_i)_{\boldsymbol{j}}^n}{\Omega(\Delta x)^2}\right) + \Delta t^n \alpha_{i,4}\right] C_{i,4}(\{p_{\boldsymbol{j}}^n\}_{\boldsymbol{j}}).$$

Guaranteeing that the coefficients of the $C_{i,k}$ terms are non-negative requires a sufficiently small Δt^n. In light of the bound (S25), it suffices for Δt^n to satisfy, for example,

$$\Delta t^n \left[\|h\|_\infty + \frac{R_\infty}{\Delta x}\left(\frac{1}{\Omega \Delta x} + 4|\mathcal{I}|\right)\right] \leq 1, \qquad \text{for} \qquad R_\infty = \sum_{i\in\mathcal{I}} \|r_i\|_\infty. \tag{S29}$$

Condition (S29) guarantees that, for each discrete state, the scheme is monotone with respect to the homogeneous terms. The terms in the square brackets in (S29) have direct interpretations: the first term represents the zeroth order exponential contributions of h from (S23) and the second term represents the contributions from the higher order differential terms. This latter term comprises two contributions in the round brackets, the first comes from the second-order term and the second from the first-order terms.

2 Code example

In this appendix, we illustrate the Python implementation of the reaction networks (6) and (7), which comprise reactions, non-local bursting, and discrete states. Each network is reproduced alongside the code required to simulate the evolution of the distribution in Table A.

The implementation of each network in the Flips software is a straightforward encoding of the network diagram: mRNA molecules are represented by the label `'m'`, protein molecules by the label `'p'`. A `solver` object is created with the network structure, reactions are separated into those governing continuum species and those governing discrete states (for which a finite truncation is specified). Each reaction includes three pieces of information: the reactants, the products, and the reaction rate. Additionally, since we follow models in the limit as $\Omega \to \infty$, the diffusion is eliminated by setting `diffusion=0`. Finally, an initial distribution is set, the distribution is evolved until some time, and the result plotted.

The interface is a direct translation of the network diagram, and therefore accessible to the non-technical user. Simultaneously, options for the advanced user allow fine tuning of the state and time discretisation (see [2]).

(i) Reaction network (6):

$$\begin{aligned} \varnothing &\xrightarrow{\text{Hill(Protein)}} \text{mRNA},\\ \text{mRNA} &\xrightarrow{\gamma B} \text{mRNA} + \text{Protein},\\ \text{mRNA} &\xrightarrow{\gamma} \varnothing,\\ \text{Protein} &\xrightarrow{\gamma_0} \varnothing. \end{aligned}$$

Python code:

```
crn =solver([
   [{'m':1}, {'m':1,'p':1}, gamma*B],
   [{'p':1}, {}, gamma0],
], discrete_truncs={'m':2}, discrete_reactions=[
   [{}, {'m':1}, Hill('p', n=n, shift=r0, coeff=r1)],
   [{'m':1}, {}, gamma],
], diffusion=0)

crn.set_initial_conditions('uniform')
crn.solve(30)
crn.plot_p()
```

(ii) Reaction network (7):

$$\begin{aligned} \varnothing &\xrightarrow{\text{Hill(Protein)}} Z \cdot \text{Protein},\\ \text{Protein} &\xrightarrow{\gamma_0} \varnothing, \end{aligned}$$

for $Z \sim \text{Geo}(1/B)$.

Python code:

```
crn =flips.solver([
   [{}, {'p':1}, burst(Hill('p', n=n, shift=r0, coeff=r1), B)],
   [{'p':1}, {}, gamma0],
], diffusion=0)

crn.set_initial_conditions('uniform')
crn.solve(30)
crn.plot_p()
```

Table A. Python code example for the Flips solver. *Code to simulate the reaction networks (6) and (7) from the self-regulated gene expression case study.*

3 Hermite equation

In this appendix, we seek separable solutions of equation (17), which we reproduce here:

$$\frac{\partial}{\partial t}q(x,t) = G(x)q(x,t) - \frac{\partial}{\partial x}[(\lambda(x)-\mu(x))q(x,t)] + \frac{1}{2\Omega}\frac{\partial^2}{\partial x^2}[(\lambda(x)+\mu(x))q(x,t)]. \tag{S30}$$

Writing $q = T(t)X(x)$, and substituting into (S30) it follows that

$$\frac{1}{T(t)}\frac{\mathrm{d}}{\mathrm{d}t}T(t) = G(x) - \frac{1}{X(x)}\frac{\mathrm{d}}{\mathrm{d}x}[(\lambda(x)-\mu(x))X(x)] + \frac{1}{2\Omega}\frac{1}{X(x)}\frac{\mathrm{d}^2}{\mathrm{d}x^2}[(\lambda(x)+\mu(x))X(x)]. \tag{S31}$$

The left-hand side is a function only of t, while the right-hand side is a function only of x, therefore these must both be constant, taking the value of the growth rate, which we will denote r. This is sometimes called the Malthus parameter [3]. In other words, $T(t) = T(0)\mathrm{e}^{rt}$, and

$$-\frac{\mathrm{d}}{\mathrm{d}x}[(\lambda(x)-\mu(x))X(x)] + \frac{1}{2\Omega}\frac{\mathrm{d}^2}{\mathrm{d}x^2}[(\lambda(x)+\mu(x))X(x)] + (G(x)-r)X(x) = 0. \tag{S32}$$

Equation (S32) is of Sturm–Liouville form, and does not have, in general, a closed-form solution. We are not aware of a closed-form solution for birth and death functions (20) even in the linear-growth case of $G(x) = gx$. Nonetheless, we may seek an asymptotic approximation in the limit as $\Omega \to \infty$. As explored in Lunz [4], the leading-order outer solution concentrates the probability mass at the critical point x_c. There was no growth term in Lunz [4], however, while this influences the growth along the characteristics, the

characteristics themselves are not affected and thus the same singular evolution unfolds in our case. This singular perturbation is regularised within a boundary layer in the vicinity of the critical point, by considering the scaling

$$x = x_c + \xi \frac{1}{\sqrt{\Omega}} \sqrt{\frac{\lambda(x_c) + \mu(x_c)}{-(\lambda'(x_c) - \mu'(x_c))}}. \tag{S33}$$

The leading-order inner form of (S32) is then given by

$$X''(\xi) + 2\xi X'(\xi) + 2(\ell + 1)X(\xi) = 0, \tag{S34}$$

where we assume that x_c is a simple root of $\lambda - \mu$, primes denoting differentiation with respect to the argument, and the constant ℓ is given by

$$\ell = \frac{g(x_c) - r}{-(\lambda'(x_c) - \mu'(x_c))}. \tag{S35}$$

Equation (S34) may be transformed to the Hermite equation under the transformation

$$Y(\xi) = \mathrm{e}^{\xi^2} X(\xi), \tag{S36}$$

whereby

$$Y''(\xi) - 2\xi Y'(\xi) + 2\ell Y(\xi) = 0. \tag{S37}$$

We now demonstrate that admissible solutions of the Hermite equation (S37) may be found if ℓ is a non-negative integer. We seek a formal series solution of the form

$$Y(\xi) = \sum_{i=0}^{\infty} c_i \xi^i. \tag{S38}$$

Upon substituting (S38) into (S37) and matching coefficients of the powers of ξ, we obtain the recurrence relation

$$c_{i+2} = \frac{2(i - \ell)}{(i+1)(i+2)} c_i. \tag{S39}$$

The recurrence relation (S39) expresses a dependence of coefficients on those indexed of the same parity. Therefore, each coefficient c_i may be expressed as a function of either c_0 or c_1, for i even or odd, respectively. The values for c_0 and c_1 may be determined by conditions at, say, the origin:

$$Y(0) = c_0, \qquad\qquad Y'(0) = c_1. \tag{S40}$$

We argue, on physical grounds, that we expect the leading-order inner solution to be symmetric about the origin, whereby $Y'(0) = 0 = c_1$, and thus all odd coefficients vanish. For a non-negative integer $\ell \geq 0$, we see from (S39) that $c_\ell = 0$, from which it follows that $c_{\ell+2j} = 0$ for all $j \geq 0$. Thus the series terminates at ℓth order to give a polynomial. These polynomials are called Hermite polynomials. Further properties of the Hermite polynomials and connections to other special functions are discussed in Arfken et al. [5]. For many applications, this family of polynomial solutions is the only admissible solution, as other values of ℓ lead to solutions that are not polynomially bounded. However, in our case the justification must be slightly stronger, since from (S36) we see that Y can grow in the far field and X may still vanish. Since the outer solution concentrates probability mass in the vicinity of the stable equilibrium, we expect it to be zero to all orders at

asymptotically large time. On this basis, we now demonstrate that solutions for values of ℓ other than the non-negative integers are inadmissible.

Consider the series solution in Y for $0 < \ell \notin \mathbb{Z}$, and denote the smallest odd integer larger than ℓ by $2L-1$. Since only even indices c_i are nonzero, it follows that

$$Y(\xi) = \sum_{i=0}^{\infty} c_{2i}\xi^{2i} = \sum_{i=0}^{\infty} \frac{d_i(\xi^2)^i}{i!}, \qquad \text{for} \qquad d_i = c_{2i}i!. \tag{S41}$$

We bound d_i by noting from (S39) and (S41) that, for $i > L$,

$$\begin{aligned}
|d_i| &= \left|\frac{2^i(2i-2-\ell)(2i-4-\ell)\cdots(2-\ell)(-\ell)c_0 i!}{(2i)!}\right| \\
&> \left|\frac{2^i(2i-2-(2L-1))(2i-4-(2L-1))\cdots(2-(2L-1))(-(2L-1))c_0 i!}{(2i)!}\right| \\
&= \frac{2^i i!(2i-1-2L)(2i-3-2L)\cdots(3)(1)}{(2i)!}\left|(-1)(-3)\cdots(-(2L-1))c_0\right| \\
&= \frac{1}{(2i-1)(2i-3)\cdots(2i+1-2L)}\left|(-1)^L(2L-1)!!c_0\right| \\
&= \frac{1}{(i+1)(i+2)\cdots(i+L)}\frac{(i+1)(i+2)\cdots(i+L)}{(2i-1)(2i-3)\cdots(2i+1-2L)}(2L-1)!!|c_0| \\
&> \frac{1}{(i+1)(i+2)\cdots(i+L)}C_L,
\end{aligned} \tag{S42}$$

where $C_L = (2L-1)!!|c_0|/2^L$ and is independent of i. We thus deduce that

$$\begin{aligned}
|Y(\xi)| + \sum_{i=0}^{L} \frac{|d_i|(\xi^2)^i}{i!} &\geq \left|Y(\xi) - \sum_{i=0}^{L}\frac{d_i(\xi^2)^i}{i!}\right| \\
&> \sum_{i=L+1}^{\infty} \frac{C_L(\xi^2)^i}{(i+L)!} \\
&= \sum_{i=0}^{L}\left(-\frac{C_L}{(i+L)!}\right)(\xi^2)^i + \frac{C_L}{\xi^{2L}}\sum_{i=0}^{\infty}\frac{(\xi^2)^{i+L}}{(i+L)!} \\
&= \sum_{i=-L}^{L} D_i(\xi^2)^i + \frac{C_L}{\xi^{2L}}\,\mathrm{e}^{\xi^2},
\end{aligned} \tag{S43}$$

for constants D_i. Substituting the bound (S43) into (S36), we find that

$$|X(\xi)| > \mathrm{e}^{-\xi^2}\sum_{i=-L}^{L}\hat{D}_i(\xi^2)^i + \frac{C_L}{\xi^{2L}}, \tag{S44}$$

for other constants $\hat{D}_i$. The second term on the right-hand side of (S44) is not beyond all orders for any L, therefore, such a solution X cannot match the outer solution and we neglect it. For $\ell < 0$, Y diverges more rapidly in the far field, since $Y(\xi;\ell)$ is monotonically increasing with respect to $\ell \notin \{0 \leq n \in \mathbb{Z}\}$. In fact, for $\ell = -1$ it turns out that $Y(\xi) = \mathrm{e}^{\xi^2}$, and X is constant. In summary, only solutions for non-negative integer ℓ are admissible.

The growth rates r_ℓ corresponding to mode ℓ are given by

$$r_\ell = g(x_c) + \ell(\lambda'(x_c) - \mu'(x_c)). \tag{S45}$$

The sequence r_ℓ is monotonically decreasing (since at a stable equilibrium $\lambda' - \mu' < 0$), therefore, for large times, the $\ell = 0$ mode will be dominant, dictating both the functional form and the growth rate. We thus deduce that the large-time behaviour will be described by constant $Y(\xi)$, and thus

$$X(x) = C\mathrm{e}^{-(x-x_c)^2/(a\Omega)}, \tag{S46}$$

where C is a normalisation constant, given by $C = 1/\sqrt{a\Omega\pi}$ for X to be a probability density. The profile is a Gaussian centred at $x = x_c$ of width $\mathcal{O}(1/\sqrt{a\Omega})$. The growth rate is approximated by r_0, namely

$$r_0 = G(x_c) = g(1 - 1/\Lambda). \tag{S47}$$

The last equality is for the specific forms $G(x) = gx$ and the functions in (20). Defining the shorthand

$$P_0 = \int_0^\infty q(x,t)\,\mathrm{d}x, \tag{S48}$$

and integrating (18), we find that

$$\frac{P_0'(t)}{P_0(t)} = \frac{\int_0^\infty G(x)q(x,t)\,\mathrm{d}x}{\int_0^\infty q(x,t)\,\mathrm{d}x}, \tag{S49}$$

that is, the growth rate is given by the G-moment of the q density. For the separable solution $q = TX = \mathrm{e}^{r_0 t}X(x)$, we see that

$$\frac{P_0'(t)}{P_0(t)} = \frac{1}{T}\frac{\mathrm{d}T}{\mathrm{d}t} = r_0, \tag{S50}$$

from which it follows that the G-moment of p_0 is precisely r_0.

4 Fragmentation models

In this appendix, we discuss analytical and numerical aspects of the growth–fragmentation equation (24), which we rewrite here:

$$\frac{\partial}{\partial t}\rho(x,t) = -\frac{\partial}{\partial x}[g(x,t)\rho(x,t)] - B(x,t)\rho(x,t) + \int_x^\infty b(y,x,t)\rho(y,t)\,\mathrm{d}y. \tag{S51}$$

First, we demonstrate that ensuring the fragmentation splits one cell into two while conserving volume is achieved by imposing the constraints [3]

$$2B(x,t) = \int_0^x b(x,z,t)\,\mathrm{d}z, \qquad xB(x,t) = \int_0^x zb(x,z,t)\,\mathrm{d}z, \tag{S52}$$

respectively. Consistency further requires that the rate (per unit size) of cell division for cells of size y into cells of size x and $y - x$ is equal, that is,

$$b(y,x,t) = b(y,y-x,t). \tag{S53}$$

From (S51), we find that the rate of change of the number of cells in the system is governed by

$$\begin{aligned}\frac{\mathrm{d}}{\mathrm{d}t}\int_0^\infty \rho(x,t)\,\mathrm{d}x = &-\int_0^\infty \frac{\partial}{\partial x}[g(x,t)\rho(x,t)]\,\mathrm{d}x \\ &-\int_0^\infty B(x,t)\rho(x,t)\,\mathrm{d}x + \int_0^\infty \int_x^\infty b(y,x,t)\rho(y,t)\,\mathrm{d}y\,\mathrm{d}x.\end{aligned} \tag{S54}$$

The first term on the right-hand side of (S54) vanishes due to the no-flux and vanishing far-field conditions. By changing the order of integration and using the first condition (S52), we find that

$$\begin{aligned}\frac{\mathrm{d}}{\mathrm{d}t}\int_0^\infty \rho(x,t)\,\mathrm{d}x &= -\int_0^\infty B(x,t)\rho(x,t)\,\mathrm{d}x + \int_0^\infty \rho(y,t)\int_0^y b(y,x,t)\,\mathrm{d}x\,\mathrm{d}y \\ &= \int_0^\infty B(x,t)\rho(x,t)\,\mathrm{d}x. \end{aligned} \tag{S55}$$

The rate of change of volume in the system is governed by

$$\begin{aligned}\frac{\mathrm{d}}{\mathrm{d}t}\int_0^\infty x\rho(x,t)\,\mathrm{d}x = &-\int_0^\infty x\frac{\partial}{\partial x}[g(x,t)\rho(x,t)]\,\mathrm{d}x \\ &-\int_0^\infty xB(x,t)\rho(x,t)\,\mathrm{d}x + \int_0^\infty\int_x^\infty xb(y,x,t)\rho(y,t)\,\mathrm{d}y\,\mathrm{d}x. \end{aligned} \tag{S56}$$

By changing the order of integration in the final term on the right-hand side of (S56) and using the second condition (S52), we find that the second and third terms cancel, thus

$$\frac{\mathrm{d}}{\mathrm{d}t}\int_0^\infty x\rho(x,t)\,\mathrm{d}x = -\int_0^\infty x\frac{\partial}{\partial x}[g(x,t)\rho(x,t)]\,\mathrm{d}x = \int_0^\infty g(x,t)\rho(x,t)\,\mathrm{d}x, \tag{S57}$$

where we again used the no-flux and vanishing far-field conditions. In fact, here we need $\rho(x,t) = o\left([xg(x,t)]^{-1}\right)$ as $x \to \infty$.

The calculations confirm the physical features of the model: the rate of change of the number of cells (S55) is given by the average rate of fragmentation B, but not explicitly dependent of the growth g, while the rate of change of volume (S57) is given by the average growth rate g, but not explicitly dependent of fragmentation.

Given a kernel b, we may derive B via one of the conditions in (S52), however, the other condition in (S52) as well as condition (S53) still constrain b in a highly non-trivial way. With the aim of simplifying the fragmentation description, as well as bringing it in line with the framework based on system (5), we introduce a new representation of the fragmentation function b (from which we will derive B) that will decouple the constraints without loss of generality. We define

$$b(y,x,t) = 2r(y,t)f_y(x,t), \tag{S58}$$

where r is the fragmentation rate of cells of size y, and f_y is the probability density that the fragmentation from size y produces, for a given daughter cell, a cell of size x. We define the corresponding cumulative probability function F_y via

$$F_y(x,t) = \int_0^x f_y(z,t)\,\mathrm{d}z, \tag{S59}$$

and note that

$$F_y(y,t) = 1. \tag{S60}$$

We satisfy the symmetry condition (S53) by requiring that

$$f_y(x,t) = f_y(y-x,t). \tag{S61}$$

We may satisfy conditions (S52) by requiring that

$$2B(x,t) = \int_0^x 2r(x,t)f_x(z,t)\,\mathrm{d}z = 2r(x,t), \tag{S62}$$

and

$$xB(x,t) = \int_0^x 2zr(x,t)f_x(z,t)\,\mathrm{d}z = 2r(x,t)\int_0^x zf_x(z,t)\,\mathrm{d}z$$
$$= 2r(x,t)\left[x - \int_0^x F_x(z,t)\,\mathrm{d}z\right], \tag{S63}$$

where the last equality comes from integrating by parts and using (S60). Upon substituting (S62) into (S63), the constraint takes the form

$$\int_0^x F_x(z,t)\,\mathrm{d}z = \frac{x}{2}. \tag{S64}$$

We proceed to show how constraint (S64) is satisfied by the representation (S58). From the probability property (S60) and the symmetry condition (S61) we see that

$$F_x(z,t) = \int_0^z f_x(s)\,\mathrm{d}s = \int_0^z f_x(x-s)\,\mathrm{d}s = \int_{x-z}^x f_x(s)\,\mathrm{d}s = F_x(x,t) - F_x(x-z,t)$$
$$= 1 - F_x(x-z,t), \tag{S65}$$

from which it follows that

$$\begin{aligned}\int_0^x F_x(z,t)\,\mathrm{d}z &= \int_0^{x/2} F_x(z,t)\,\mathrm{d}z + \int_{x/2}^x F_x(z,t)\,\mathrm{d}z \\ &= \int_0^{x/2} F_x(z,t)\,\mathrm{d}z + \int_{x/2}^x 1 - F_x(x-z,t)\,\mathrm{d}z \\ &= \int_0^{x/2} F_x(z,t)\,\mathrm{d}z + \frac{x}{2} - \int_0^{x/2} F_x(z,t)\,\mathrm{d}z \\ &= \frac{x}{2}.\end{aligned} \tag{S66}$$

To recap, by introducing the representation (S58) for b based on a decomposition into a rate and a symmetric probability density, we trivially satisfy both conditions in (S52) and condition (S53). The fragmentation rate B is equivalent to the rate r in this representation (and to the rate f in the ACME formulation (5)). Importantly, this representation expresses the kernel b in terms of identifiable quantities. In particular, the practitioner needs to specify the rate r at which fragmentation occurs for each cell size, and the distribution of fragments f_y. The kernel $\mathcal{B}$ in the ACME formulation (5) is given by $2f_y$, and is thus twice a probability density.

There are additional advantages to this representation. This formulation allows for a straightforward description of self-similar fragmentation kernels, that is, the dependence of f_y on y is only a rescaling, namely

$$f_y(x,t) = \frac{1}{y}f(x/y,t), \tag{S67}$$

where $f(z,t)$ is a density on $z \in (0,1)$. Symmetric and asymmetric cell division, for example, is commonly modeled with size distributions in proportion with the original cell [3], in line with this description. Moreover, it is easy to incorporate Dirac delta functions within f_y since the finite-volume scheme is built via integrating over finite regions of the state space. Therefore, the cumulative representation of f_y via F_y suffices, and this is a well-defined function. To see this, we detail the discretisation of the fragmentation operator.

As with bursts, for each volume centred at $i\Delta x$ we subtract the propensity of fragmenting to within the volume centred at $j\Delta x$ for $0 \leq j < i$ and add that propensity to the j volume, thereby achieving conservation of volume. Note that in this paragraph we have used the word "volume" to refer to what is normally called a "cell", so as not to confuse the use of the word "cell" elsewhere in this paper referring to the biological entity. The probability of fragmenting from j to i is given by

$$\begin{aligned}\mathbb{P}(\text{fragment } j \to i) &= \int_{(j-1/2)\Delta x}^{(j+1/2)\Delta x} \int_{(i-1/2)\Delta x}^{(i+1/2)\Delta x} f_y(x,t)\,\mathrm{d}x\,\mathrm{d}y \\ &= \int_{(j-1/2)\Delta x}^{(j+1/2)\Delta x} F_y((i+1/2)\Delta x, t) - F_y((i-1/2)\Delta x, t)\,\mathrm{d}y. \end{aligned} \tag{S68}$$

The probability (S68) is expressed via its cumulative density. Further simplification is possible, for example, in the self-similar case,

$$\mathbb{P}(\text{fragment } j \to i) = \frac{1}{\Delta x}\int_0^1 F\left(\frac{i+1/2}{j-1/2+Y}, t\right) - F\left(\frac{i-1/2}{j-1/2+Y}, t\right)\mathrm{d}Y. \tag{S69}$$

If the distribution is only Dirac masses, for example, time-invariant asymmetric cell division at size fractions c and $1-c$ for $c \in (0,1)$, then $f(x,t) = [\delta(x-c)+\delta(x-1+c)]/2$, in which case (S69) further simplifies to

$$\begin{aligned}\mathbb{P}(\text{fragment } j \to i) &= \frac{1}{2\Delta x}\int_0^1 \mathbb{1}_{(i+1/2)/c-(j-1/2)>Y} + \mathbb{1}_{(i+1/2)/(1-c)-(j-1/2)>Y} \\ &\qquad - \mathbb{1}_{(i-1/2)/c-(j-1/2)>Y} - \mathbb{1}_{(i-1/2)/(1-c)-(j-1/2)>Y}\,\mathrm{d}Y \\ &= \frac{1}{2\Delta x}\Big[Q((i+1/2)/c-(j-1/2)) + Q((i+1/2)/(1-c)-(j-1/2)) \\ &\qquad - Q((i-1/2)/c-(j-1/2)) - Q((i-1/2)/(1-c)-(j-1/2))\Big],\end{aligned} \tag{S70}$$

where $\mathbb{1}_{x>0}$ is the Heaviside function and Q is the integral

$$Q(c) = \int_0^1 \mathbb{1}_{x<c}\,\mathrm{d}x = \min(1, \max(0, c)). \tag{S71}$$

The formula (S70) accelerates construction of the scheme since no integrals need to be computed, and demonstrates how Dirac delta functions are accurately captured in the finite volume discretisation. The fragmentation probability is multiplied by the fragmentation rate r to give the local fragmentation propensity, and probability mass is transferred from volume j in proportion to the propensity, to volume i with twice this proportion to account for the cell creation (this is the factor of 2 in (S58)).

5 Explicit growth–fragmentation solutions

In this appendix, we compile analytical solutions of growth–fragmentation equations for benchmark use. We begin by considering the growth-free problem considered in Rooney et al. [6], namely

$$\frac{\partial}{\partial t}\rho(x,t) = \int_x^\infty 2ay^{k-1}\rho(y,t)\,\mathrm{d}y - ax^k\rho(x,t). \tag{S72}$$

The equation (S72) corresponds to the growth–fragmentation model (24) with no growth $g \equiv 0$ and fragmentation kernel $b(y,x) = 2ay^{k-1}$, from which we deduce, via (S52), that $B(x) = ax^k$.

We refer the reader to Rooney et al. [6] for a step-by-step solution of (S72). In brief, we seek a similarity solution of the form

$$\rho(x,t) = x^{\alpha} h(\eta), \qquad \eta = xt^{\beta}. \tag{S73}$$

It can be shown that the scalings must satisfy $\alpha = -2$ and $\beta = 1/k$. The equation (S72) may then be reduced to an ODE for h, admitting the solution

$$\rho(x,t) = x^{-2} h(\eta) = c(at)^{2/k} \mathrm{e}^{-atx^k}, \tag{S74}$$

where c is an arbitrary constant.

We now employ a change of variables presented in Cáceres et al. [7] which transforms the solution of the general growth-free equation

$$\frac{\partial}{\partial t}\rho(x,t) = \int_x^{\infty} b(y,x)\rho(y,t)\,\mathrm{d}y - B(x)\rho(x,t), \tag{S75}$$

such as (S72), to the solution of a growth–fragmentation equation with non-uniform growth and uniform degradation. We assume that the fragmentation kernel is time-invariant homogeneous of degree $\gamma - 1$ for $\gamma > 0$, that is,

$$b(sy, sx) = s^{\gamma-1} b(x,y), \tag{S76}$$

for any $s > 0$. For equation (S72) the kernel is homogeneous with $\gamma = k$.

We now define

$$\hat{\rho}(x,t) = \mathrm{e}^{-2t}\rho(\mathrm{e}^{-t}x, \mathrm{e}^{\gamma t} - 1), \tag{S77}$$

and look for an equation governing the time evolution of $\hat{\rho}$ by taking partial derivatives. First with respect to x, we find that

$$\frac{\partial}{\partial x}\hat{\rho}(x,t) = \mathrm{e}^{-3t}\frac{\partial \rho}{\partial x}(\mathrm{e}^{-t}x, \mathrm{e}^{kt} - 1). \tag{S78}$$

Then, using the dynamics (S72), and (S78), we see that

$$\begin{aligned}\frac{\partial}{\partial t}\hat{\rho}(x,t) &= -2\mathrm{e}^{-2t}\rho(\mathrm{e}^{-t}x, \mathrm{e}^{kt}-1) - \mathrm{e}^{-3t}x\frac{\partial \rho}{\partial x}(\mathrm{e}^{-t}x, \mathrm{e}^{kt}-1) + k\mathrm{e}^{(k-2)t}\frac{\partial \rho}{\partial t}(\mathrm{e}^{-t}x, \mathrm{e}^{kt}-1)\\ &= -2\hat{\rho}(x,t) - x\frac{\partial}{\partial x}\hat{\rho}(x,t) + k\mathrm{e}^{(k-2)t} \times\\ &\qquad \left[\int_{\mathrm{e}^{-t}x}^{\infty} b(y, \mathrm{e}^{-t}x, \mathrm{e}^{kt}-1)\rho(y, \mathrm{e}^{kt}-1)\,\mathrm{d}y - B(\mathrm{e}^{-t}x)\rho(\mathrm{e}^{-t}x, \mathrm{e}^{kt}-1)\right].\end{aligned} \tag{S79}$$

Upon substituting (S77), changing variables, and using homogeneity assumption (S75), the square brackets on the right-hand side of (S79) may be expressed as

$$\begin{aligned}&\int_{\mathrm{e}^{-t}x}^{\infty} b(y, \mathrm{e}^{-t}x)\rho(y, \mathrm{e}^{kt}-1)\,\mathrm{d}y - B(\mathrm{e}^{-t}x)\rho(\mathrm{e}^{-t}x, \mathrm{e}^{kt}-1)\\ &\qquad = \mathrm{e}^{t}\int_x^{\infty} b(\mathrm{e}^{-t}z, \mathrm{e}^{-t}x)\hat{\rho}(z,t)\,\mathrm{d}z - \mathrm{e}^{(2-k)t}B(x)\hat{\rho}(x,t)\\ &\qquad = \mathrm{e}^{(2-k)t}\left[\int_x^{\infty} b(z,x)\hat{\rho}(z,t)\,\mathrm{d}z - B(x)\hat{\rho}(x,t)\right],\end{aligned} \tag{S80}$$

whereby we see that $\hat{\rho}$ satisfies the growth–fragmentation equation

$$\frac{\partial}{\partial t}\hat{\rho}(x,t) = -\frac{\partial}{\partial x}\left[x\hat{\rho}(x,t)\right] - \hat{\rho}(x,t) + k\left[\int_x^{\infty} b(z,x)\hat{\rho}(z,t)\,\mathrm{d}z - B(x)\hat{\rho}(x,t)\right]. \tag{S81}$$

Equation (S81) captures growth linear in the cell size x and uniform decay. Using b (and thus B) from equation (S72) means that $\hat{\rho}$ describes a process that inherits an analogous fragmentation behaviour occurring at k times the rate.

References

1. Kurganov A, Tadmor E. New high-resolution central schemes for nonlinear conservation laws and convection–diffusion equations. J Comput Phys. 2000;160(1):241–282.

2. Lunz D. Flips solver library. Version 0.0.2 [software]; 2021. [Available from]: `https://gitlab.inria.fr/dlunz/flips`.

3. Perthame B. Transport equations in biology. Birkhäuser Basel; 2006.

4. Lunz D. On Continuum Approximations of Discrete-State Markov Processes of Large System Size. Multiscale Modeling & Simulation. 2021;19(1):294–319. doi:10.1137/20M1332293.

5. Arfken GB, Weber HJ, Harris FE. Mathematical methods for physicists. 7th ed. Academic Press; 2012.

6. Rooney CM, Griffiths IM, Brunner C, Potter J, Wood-Lee M, Please CP. Dynamics of particle chopping in blenders and food processors. J Eng Math. 2018;112(1):119–135.

7. Cáceres MJ, Cañizo JA, Mischler S. Rate of convergence to an asymptotic profile for the self-similar fragmentation and growth-fragmentation equations. J Math Pure Appl. 2011;96(4):334–362. doi:10.1016/j.matpur.2011.01.003.
